# Supplementary material for: Tough asymmetric thermochromic ionogels via dynamic in situ phase separation for dual-modal smart optical switching
Source: Nat Commun. 2026 Mar 18;17:4124. doi: 10.1038/s41467-026-70830-4 (PMC13150026; doi:10.1038/s41467-026-70830-4)
Supplement: Supplementary file 1 — Supplementary Information [file 41467_2026_70830_MOESM1_ESM.pdf]

Supplementary Information

Tough asymmetric thermochromic ionogels via dynamic in situ phase separation for dual-modal smart optical switching

Guoli Du（杜国立）,[1] Jianing Li（李嘉宁）,[1] Changxing Wang（王昌兴）,[1] Jianing Li（李佳宁）,[1] Yayun Ning（宁雅云）,[1] Yifan Yue（岳艺凡）,[1] Yuechi Xie（谢岳池）,[1] Sen Yang（杨森）,[1]* Xuegang Lu（卢学刚）[1]*

[1]School of Physics, MOE Key Laboratory for Nonequilibrium Synthesis and Modulation of Condensed Matter, Xi'an Jiaotong University, Xi'an 710049, China

*Corresponding author: xglu@mail.xjtu.edu.cn; yangsen@mail.xjtu.edu.cn

This file includes

Supplementary Methods

Supplementary Figures 1-49

Supplementary Tables 1-5

Supplementary Notes 1-6

Supplementary References S1-S21

All content in the main text, Supplementary Information, and Supplementary Movies that features Xi'an Jiaotong University's logo, emblem, motto, and buildings is used with permission. All schematic illustrations in the main text and Supplementary Information were created by the authors using Microsoft PowerPoint and Blender.

Supplementary Methods

Materials

The monomers n-butyl acrylate (BA), N-isopropylacrylamide (NIPAM), and 2-hydroxyethyl acrylate (HEA), Irgacure 1173, cross-linker ethylene glycol dimethacrylate (EGDMA) and N,N′-methylenebis (acrylamide) (MBAA), thermal initiator ammonium persulfate (APS), and accelerator N,N,N′,N′-tetramethylethylenediamine (TEMED) were purchased from Aladdin. The ionic liquid [Emim][TFSI], [Emim][BF_4], [Emim]Cl, and [Emim][$EtSO_4$] were purchased from Lanzhou Institute of Chemical Physics. The reagents used in the experiments were used as received without further purification, and the DI water used was purified by Master-Q30. Flexible graphene heating sheet with polyethylene terephthalate (PET) substrate and copper tape purchased from Shenzhen Nuanfeng Technology Co. The following materials were purchased from Alibaba: transparent sheets including glass, polymethyl methacrylate (PMMA), polyvinyl chloride (PVC), polycarbonate (PC); commercial tape including polyimide (PI), 3M VHB tape, 3M 9080 tape; and insulated foam boxes.

Preparation of pure PBA ionogels, bilayer ionogels and PNIPAM hydrogels

Pure PBA ionogels were prepared in a similar manner as ATI-B in asymmetric ionogels, except that there was no subsequent in situ light curing. Physical bilayer ionogels were prepared by preparing ionogels with ATI-B and ATI-BN as precursors, respectively, and using an external force to adhere them together and overnight.

9.2 mmol of NIPAM was dissolved in 13 mL of DI water. Sequentially, 0.26 mmol of MBAA, 0.09 mmol of APS and 0.27 mmol of TEMED were added to the solution. The

solution was stirred at 0°C for 2 h and then placed in a refrigerator at 2-4°C overnight. The resulting gel was immersed in DI water for 48 h to obtain PNIPAM hydrogel.

Computational methods

Molecular dynamics (MD) simulations were performed within Materials Studio using the Forcite module. The initial computational model comprised a simulation box measuring 3.29 nm^3, containing 10 PBA chains (each with a polymerization degree of 10) and 22 [EMIM][TFSI] ionic liquid molecules. An initial amorphous structure was generated by subjecting this model to geometry relaxation using the COMPASS II force field. Subsequently, MD simulations employing the COMPASS II force field were carried out. The system underwent NPT ensemble equilibration for 100 ps using a Nosé thermostat and barostat to achieve system equilibration. For the analysis of structural and diffusional properties, NVT ensemble simulations were conducted for 1000 ps at temperatures of either 298K or 313K, employing a time step of 1.0 fs. Interaction energies were performed using the Dmol^3 code in Materials Studio software. Van der Waals correction of Grimme's DFT-D3 model[1] was used, together with the GGA/PBE[2,3] exchange-correlation functional and a DNP basis set. Next, an energy convergence criterion of 1×10^{-5} Ha and a force convergence criterion of 0.002 Ha Å^{-1} were set in the calculation. The interaction energy (E) was calculated by the following equation:

$$E=E_{A+B}-(E_A+E_B) \tag{1}$$

where E_{A}, E_{B}, and $E_{\mathrm{A+B}}$ are the energy of the isolate A, B, and A-B complex, respectively.

Mechanical performance test

Tensile and compression tests were performed using an electronic universal testing machine (3367, Instron, USA). The tensile samples were dumbbell-shaped with a tensile area size of 20 × 35 × 2 mm and a tensile speed of 50 mm min^{-1}. Sandpaper and tape were used to secure the ionogel to the fixture during testing. The compression specimen was cylindrical with a diameter of 10 mm and a height of 5 mm, and the compression speed was 50 mm min^{-1}; the maximum strain value for the compression cycle test was set at 70%. For the lap-shear experiments, two Al sheet were used as substrate sand glue was used to bond the ionogels to them. The contact area between the ionogel and the aluminum sheet was 25 × 25 mm, and the test speed was 20 mm min^{-1}. To demonstrate the excellent interfacial strength of the ATIs, two commercial double-sided adhesives (VHB and 9080) were purchased from 3M for comparison. For adhesion testing, ATIs were cut into 20 × 100 × 2 mm blocks. They were adhered to different substrates and to avoid air bubbles at the interface, pressed using a heavy weight and left overnight. PI tapes were used as a backing to avoid stretching of the ATIs during the tests. To test impact resistance, equal sized ATIs were sandwiched between two white PVC sheets, along with tempered glass, and a 1.25 t vehicle was driven over them to assess their morphological integrity.

Surface hydrophobicity test

Surface hydrophobicity tests were performed on glass, PNIPAM hydrogel, and ATI using an optical contact angle meter (DSA100S, Kruss, Germany). The glass was ultrasonically cleaned and dried using DI water and alcohol prior to testing. Dust-free

paper was used to vacuum the water from the surface of the PNIPAM hydrogel. No additional treatment was performed on the ATI except for the use of alcohol to clean the surface of floating dust. ATIs were tested for surface droplet adhesion and antifouling ability using a mixture of toluidine blue and toner configured respectively. According to the Hamaker theory and the Young-Dupré equation[4, 5], the work of adhesion is given by the following equation:

$$W_{LS}=\gamma_L(1+cos\theta) \qquad (2)$$

where W_{LS} is the work of adhesion at the interface of the two phases, L and S represent liquids and solids, respectively; the surface tension (γ) of water at room temperature is 72 mN m^{-1}; and θ is the contact angle.

Smart cooling window test

Transmittance variations of ATIs in the 200-2500 nm wavelength range over 20-45°C were measured using a UV-Vis-NIR spectrophotometer (Lambda 950, PerkinElmer, USA) and a temperature-controlled accessory. To evaluate the cooling performance of the material, aluminum foil-covered commercial foam boxes (inside diameter: 22.8 × 12 × 14.7 cm, wall thickness: 3 cm) with a top opening size of 12 × 12 cm were used as a model house with one window. Tempered glass, ATI (attached to glass, ATI-B side close to the glass), and air (open system) were used as the windows of the model house, respectively. A xenon lamp was used to simulate sunlight to evaluate the cooling ability of the material and a thermocouple (TA612C, TASI, China) was used for temperature monitoring. When the field test of smart cooling windows was conducted, a model

house with different materials as windows was placed outdoors (Xi'an, China, May 20, 2025) and the temperature inside was monitored for 24 h. To prepare the aesthetic smart windows, the shape of the ATI was customized using the template cutting method and stained using dyes.

Energy-saving performance simulation

To evaluate the energy-saving and cooling performance of the ATI smart window, we conducted building energy simulations using the open-source software EnergyPlus (version 23.2.0). The building model was constructed using SketchUp (version Pro 2021). A typical container house model measuring 27 m (L) × 18 m (W) × 4 m (H) (6 stories) was employed (Supplementary Fig. 49). Through whole-building energy simulation, the HVAC energy savings attributable to the test sample were estimated. First, a baseline scenario was established without the ATI smart window. The HVAC system was configured to activate heating mode when the ambient temperature dropped below 16 °C, and cooling mode when the indoor temperature exceeded 26 °C. The corresponding HVAC energy consumption was computed under these conditions. Subsequently, the ATI was incorporated into the model as a window energy-saving component, maintaining the original building structure. The HVAC energy consumption was recalculated under identical operational settings. Climate data for various cities, obtained from the EnergyPlus website (https://energyplus.net/weather), were used to simulate the HVAC energy demands for both the baseline and ATI-integrated building models across different climatic conditions.

Smart projection screen test

For localized opacity display of ATIs, a patterned customized flexible graphene heating sheet was used as a Joule heating substrate, which was powered using a DC power supply (24 V). To realize an in situ global opaque display of the ATI, the ATI body can be used as a flexible Joule heater. A signal generator (FY6900, Feiyi, China) and a high voltage amplifier (ATA-2041, Aigtek, China) were used to power the ATI. The local maximum temperature of the ATI can reach 50°C when the operating frequency and voltage were 10 kHz and 180 V, respectively. Electrochemical impedance spectra (EIS) of ATIs were obtained by an electrochemical workstation (PARSTAT MS, Princeton, USA), thermoregulated using a temperature control accessory, and temperature monitored using a thermocouple. The ionic conductivity of ATI was calculated by the following equation:

$$\sigma = \frac{L}{R_b \times S} \tag{3}$$

where σ is the ionic conductivity; L is the thickness of the specimen sandwiched between the electrodes; R_b is the intrinsic resistance of the specimen; and S is the contact area between the specimen and the electrode.

An infrared thermal imager (TiS55+, Fluke, USA) was used to visualize the local heat distribution on the ATIs. When used as smart projection screens, ATIs are transformed opaque by indirect localized heating or direct Joule heating, and projections are made on their surfaces using a commercial micro-projector (w01, Wanpi, China).

Long-term stability test

High temperature and high humidity cycle test: 4 h of high temperature (60°C) followed by 4 h of high-humidity environment (humidity >90%), total of 5 cycles.

Ultraviolet test: The ATI was irradiated with UVA (365 nm, 10 mW cm^{-2}) and UVB (311 nm, 1 mW cm^{-2}) respectively at a vertical distance of 15 cm for 12 h; the ultraviolet light intensity was confirmed by a UV energy meter (HX8203, HXMETE, China).

Salt spray aging test: Prepare a 5 wt% sodium chloride aqueous solution with pH = 7. Atomize the solution using a mist humidifier (KLS-232, Kaileshi, China) to maintain an ambient humidity of >90%. Conduct a continuous 24 h aging test on ATI under these conditions.

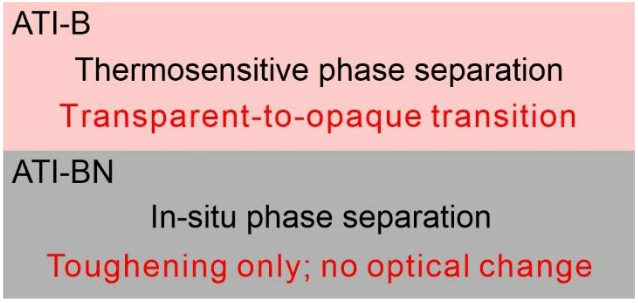

Supplementary Figure 1. The bilayer asymmetric structure consisting of ATI-B and ATI-BN within ATI, where each layer has a distinct and independent function.

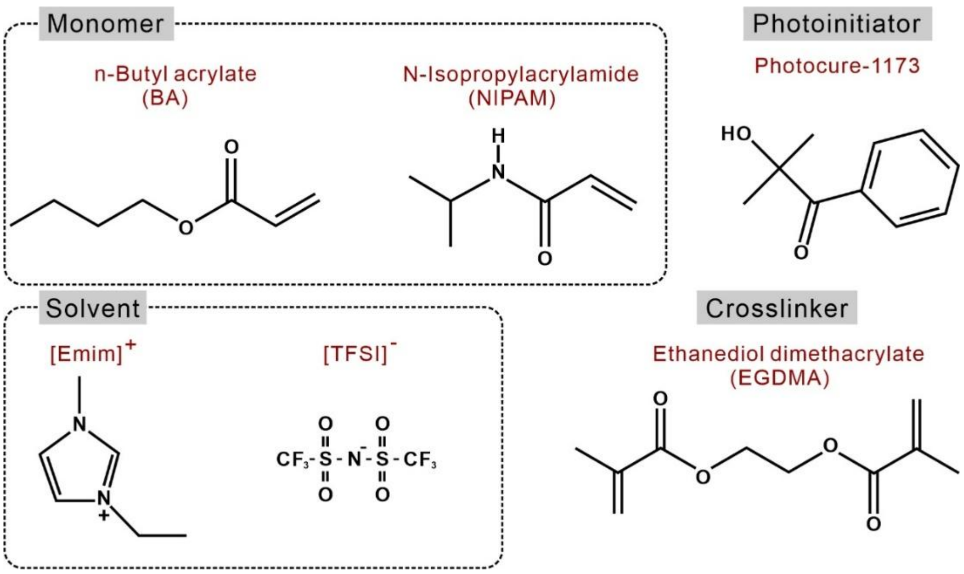

Supplementary Figure 2. Molecular structures of chemicals used in the synthesis of ATI precursors.

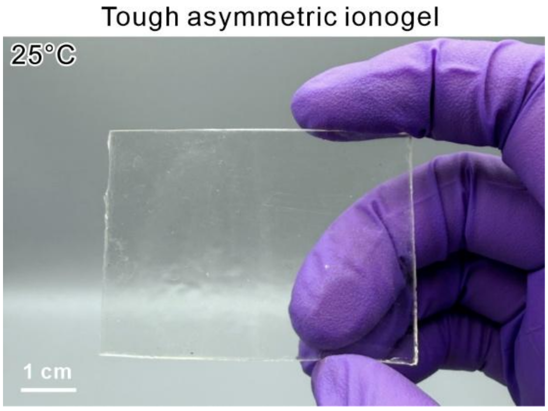

Supplementary Figure 3. Optical photograph of ATI.

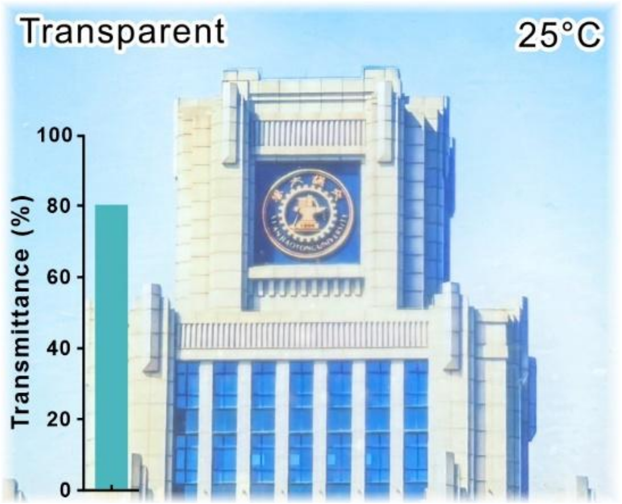

Supplementary Figure 4. Optical photograph of ATI at 25°C, inset shows the transmittance of light at 550 nm wavelength.

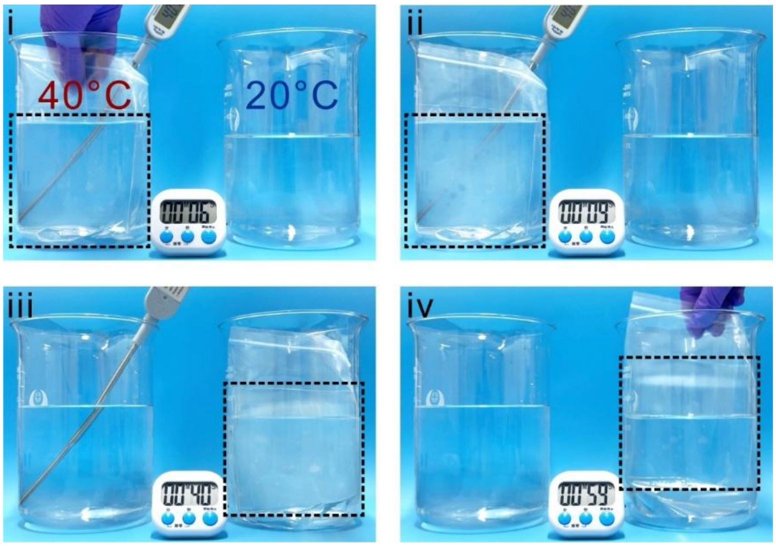

Supplementary Figure 5. The thermochromic process of ATI. Including i) immersion in hot water at 40°C; ii) an opaque transformation; iii) transfer to cool water at 20°C; and iv) return to a transparent state.

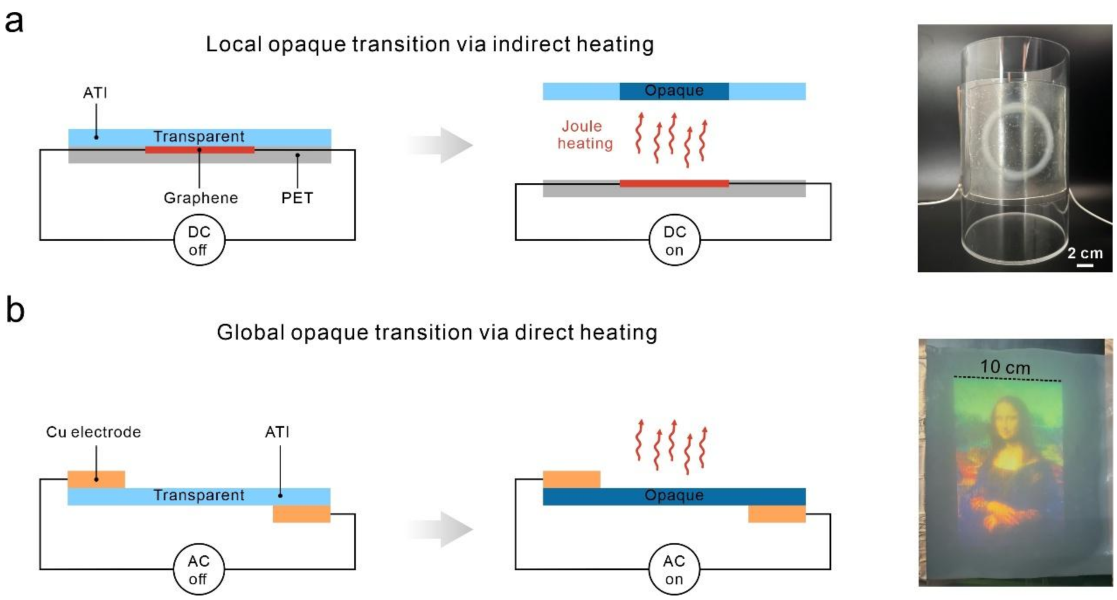

Supplementary Figure 6. Local/global opaque transitions achieved by indirect/direct Joule heating of ATI. a) Schematic of the process of indirect Joule heating of ATI using flexible graphene heating sheets (left) and local display effect (right). b) Schematic of the process of direct Joule heating of ATI using alternating current (left) and display effect of global projection screen (right, "Mona Lisa", Leonardo da Vinci, 1503-1506, public domain work).

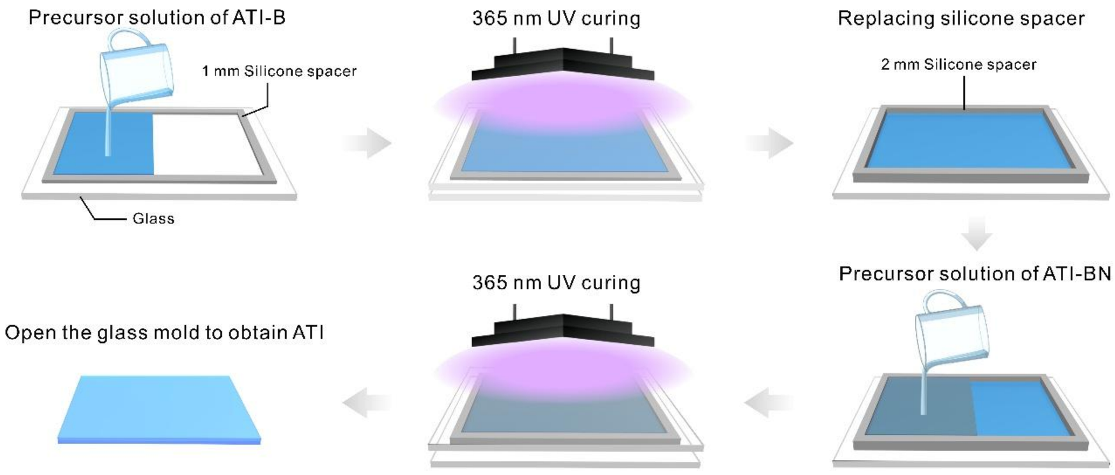

Supplementary Figure 7. Schematic of the ATI preparation process.

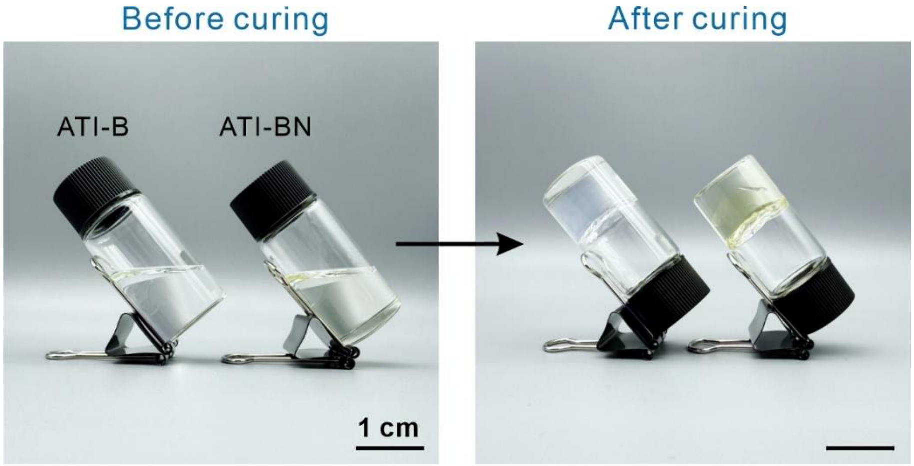

Supplementary Figure 8. Photographs of precursor solutions of ATI-B and ATI-BN before and after curing, respectively.

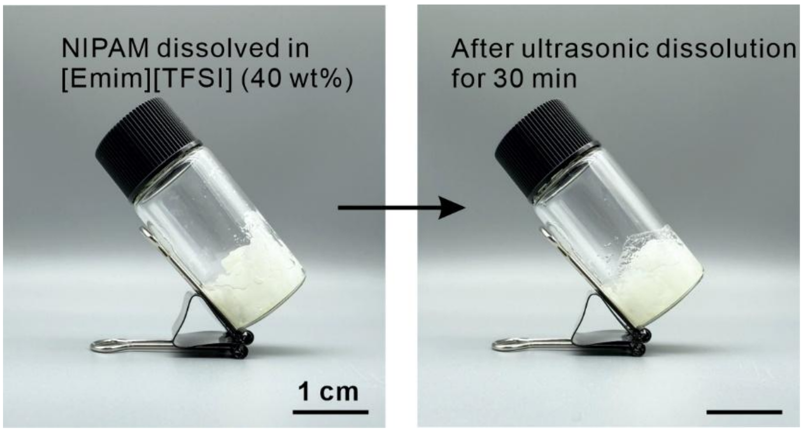

Supplementary Figure 9. Demonstration of NIPAM solubility in [Emim][TFSI].

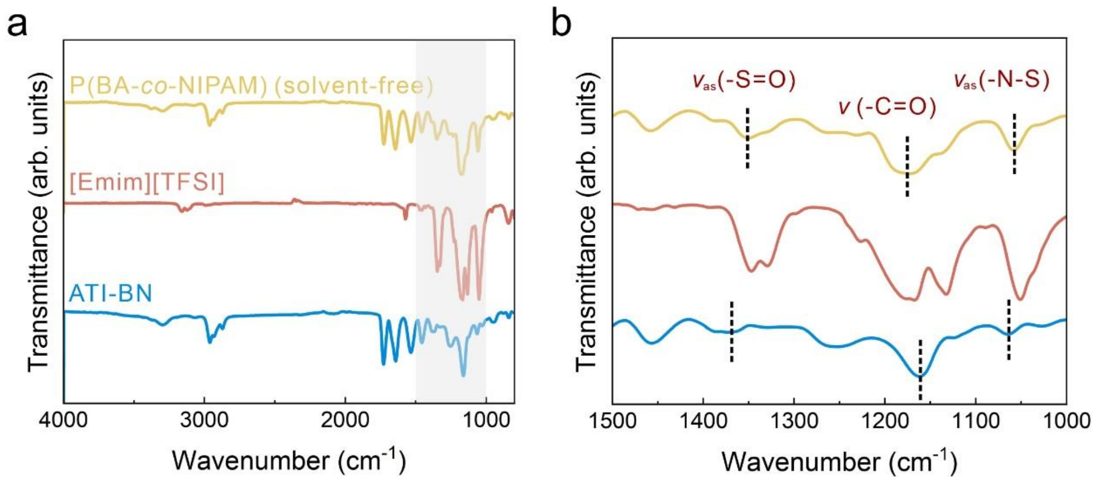

Supplementary Figure 10. FTIR spectra of solvent-free P (BA-co-NIPAM), solvent [Emim] [TFSI], and ionogel ATI-BN. a) Wavenumbers in the range of 800-4000 cm^{-1}. b) Wavenumbers in the range of 1000-1500 cm^{-1}. The same color refers to the same component.

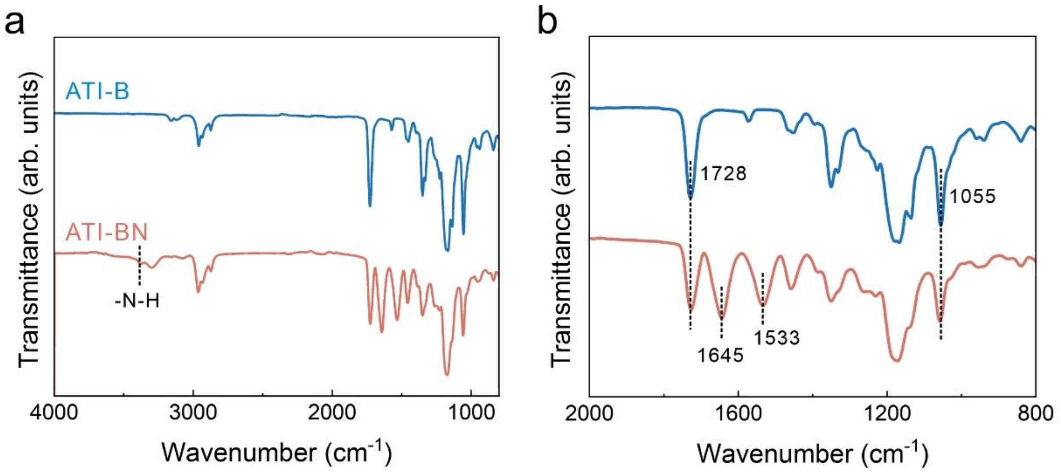

Supplementary Figure 11. FTIR spectra of ATI-B and ATI-BN. a) Wavenumbers in the range of 800-4000 cm^{-1}. b) Wavenumbers in the range of 800-2000 cm^{-1}. The same color refers to the same component.

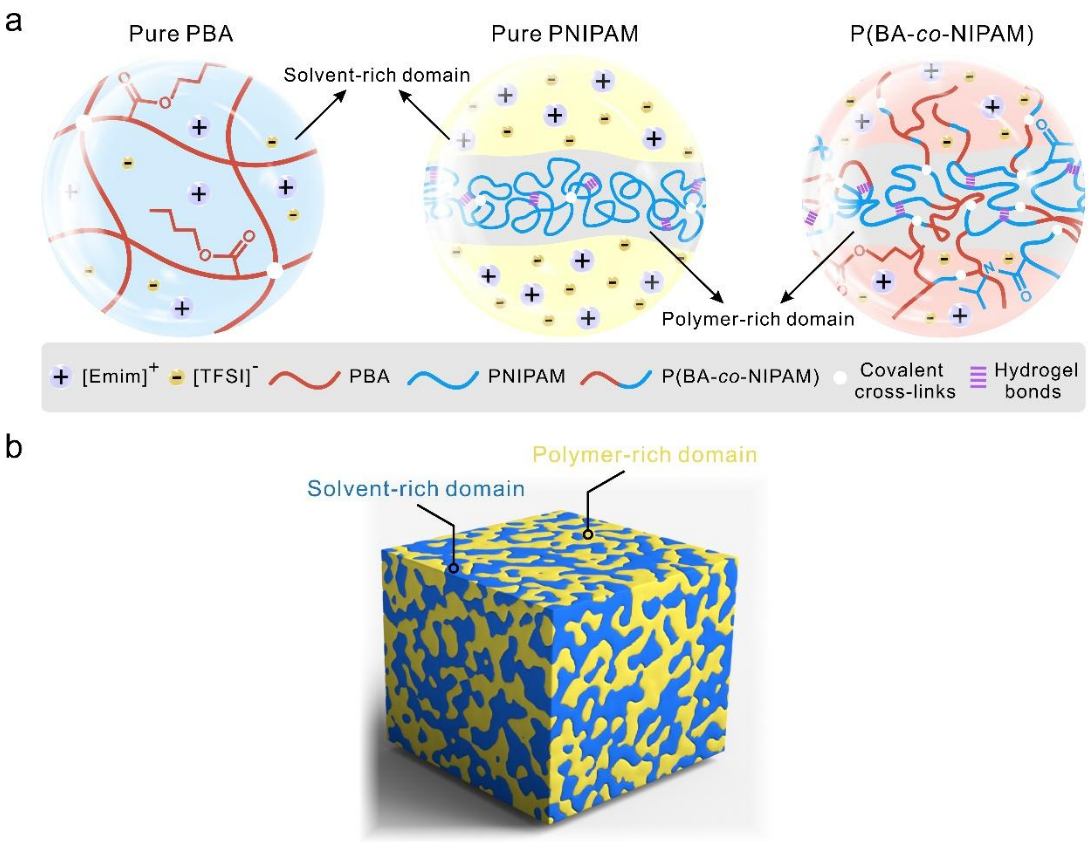

Supplementary Figure 12. Schematic of phase separation in ATI-BN. a) Homopolymer networks of PBA and PNIPAM; and copolymer networks of P(BA-co-NIPAM) in phase-separated structures. b) Bicontinuous networks in in situ phase-separated structures.

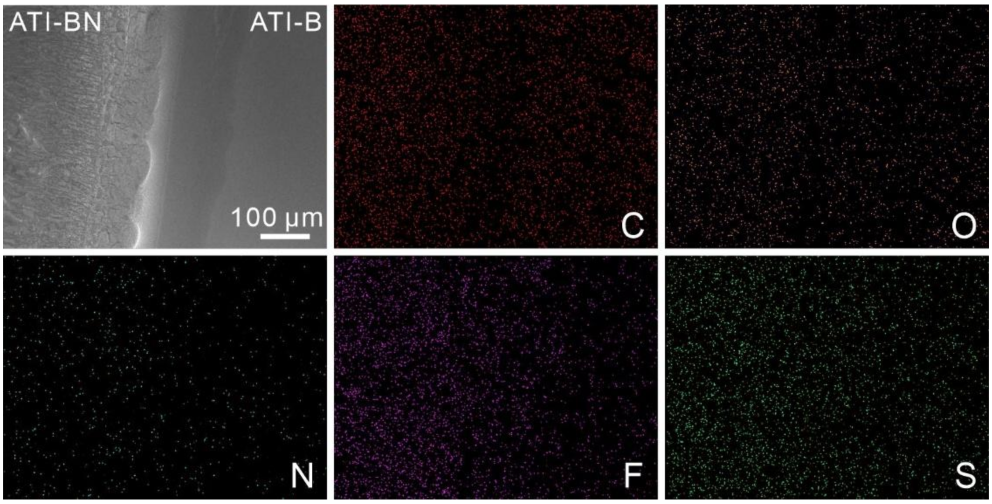

Supplementary Figure 13. Cross-section SEM image and EDS mapping of ATI.

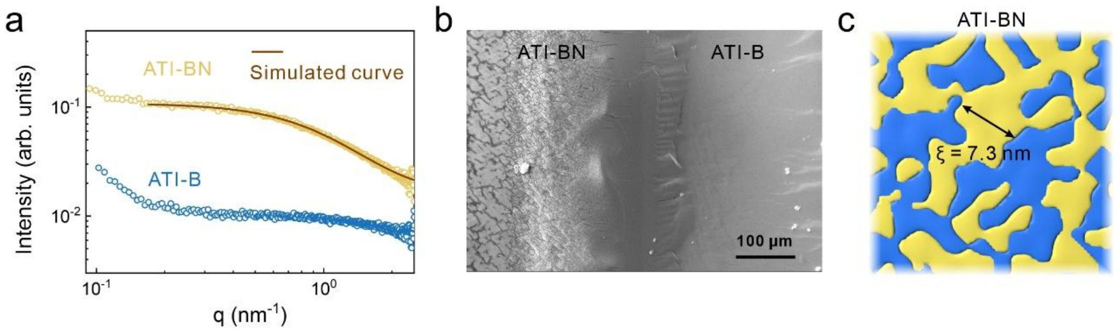

Supplementary Figure 14. Analysis of bicontinuous structure in ATI-BN. a) 1D SAXS spectra. b) SEM image at the interface of ATI, with ATI-BN (rough) on the left and ATI-B (smooth) on the right. c) The schematic illustrates the bicontinuous structure and in-suit phase-separated correlation length in ATI-BN. The characteristic size of separated phase is about 7.3 nm, which is far below the wavelengths of visible light, thereby strengthening the ionogel without changing its transparency.

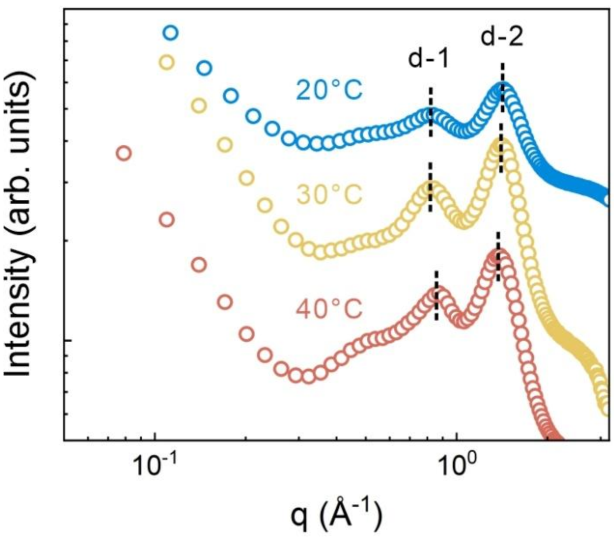

Supplementary Figure 15. 1D WAXS spectra of ATI-B.

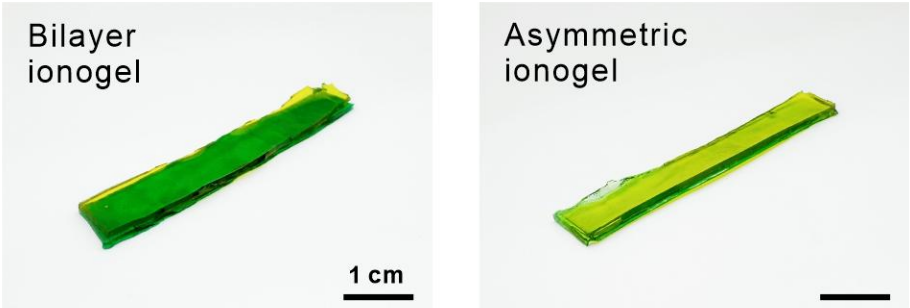

Supplementary Figure 16. Optical photographs of bilayer ionogel and asymmetric ionogel after staining treatment.

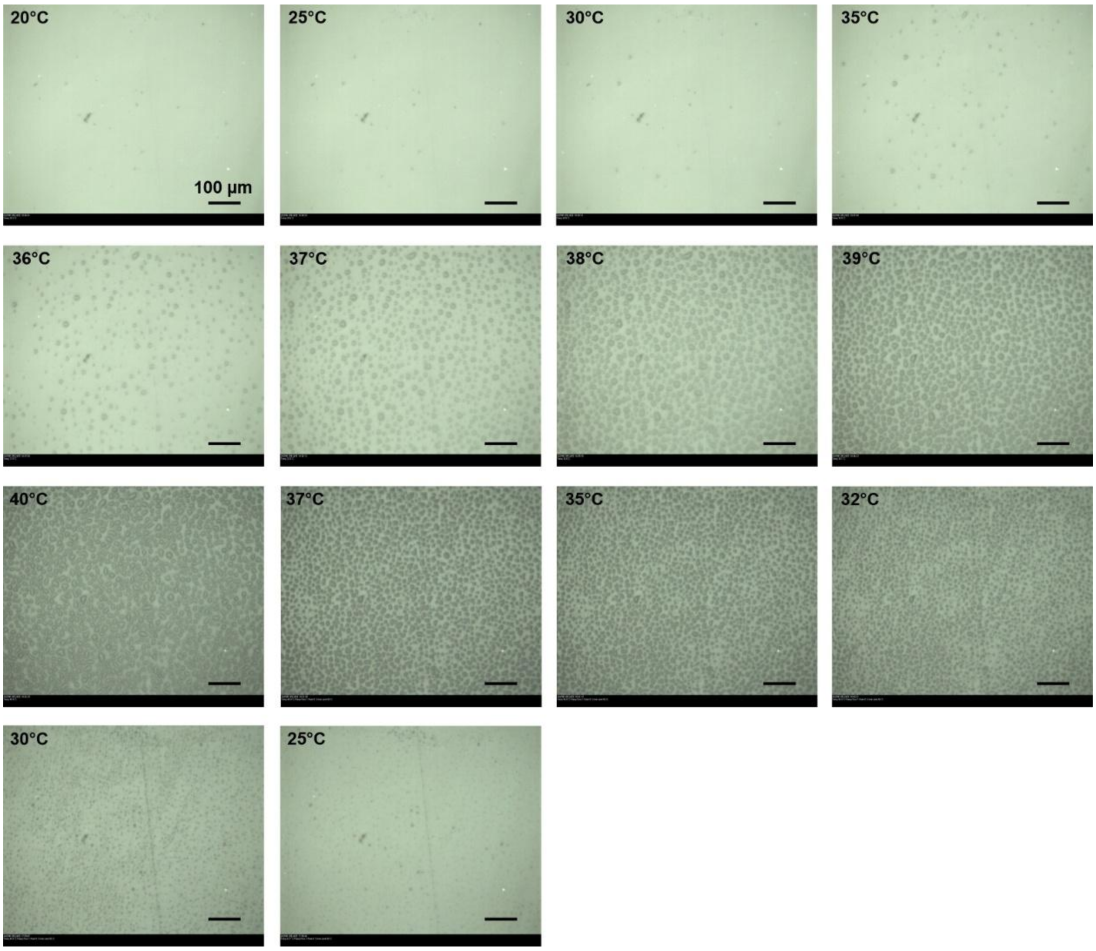

Supplementary Figure 17. Real-time observation of the thermally induced phase separation in the ATI-B layer. Selected frames from Supplementary Movie 3 showing the thermal evolution of micron-scale ionic liquid domains at the critical transition temperature. Scale bar: 100 µm.

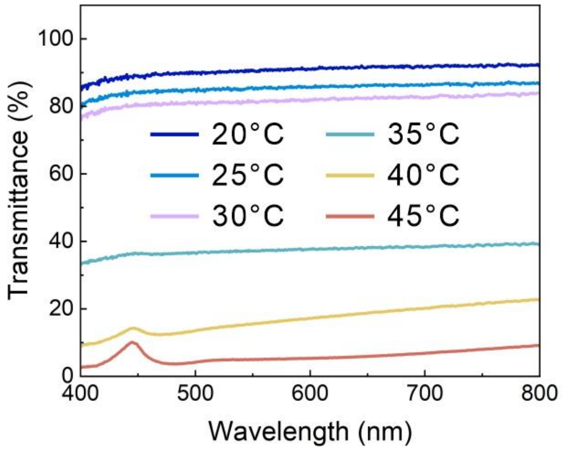

Supplementary Figure 18. The transmittance of ATI within the visible light range when the temperature varies within the range of 20-45℃.

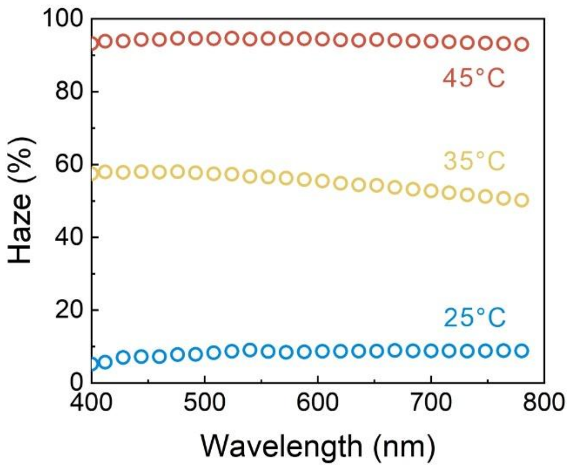

Supplementary Figure 19. Variation of haze with temperature within the visible light wavelength range.

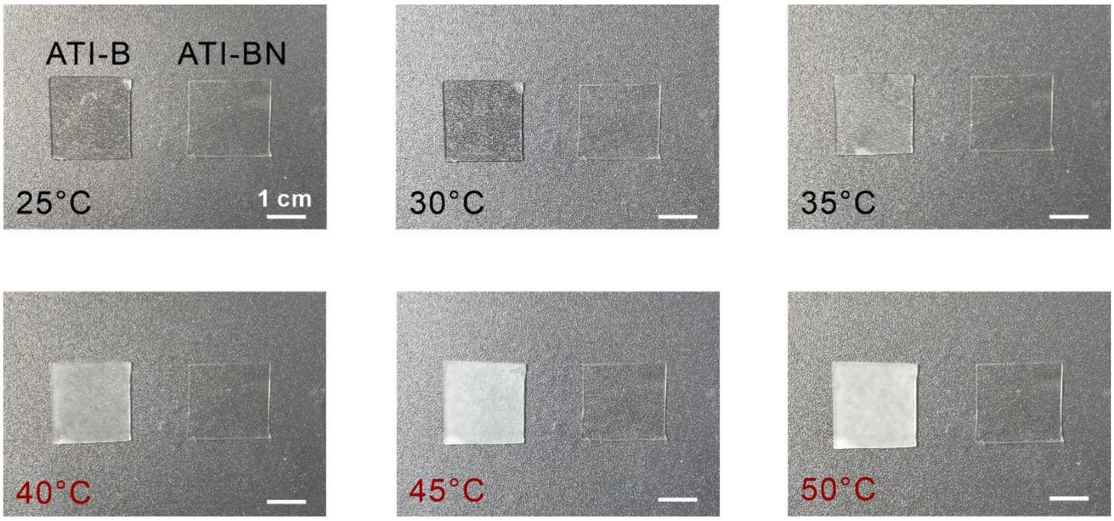

Supplementary Figure 20. The changes in optical performance observed by heating ATI-B and ATI-BN separately within the temperature range of 25–50°C. Scale bar: 1 cm.

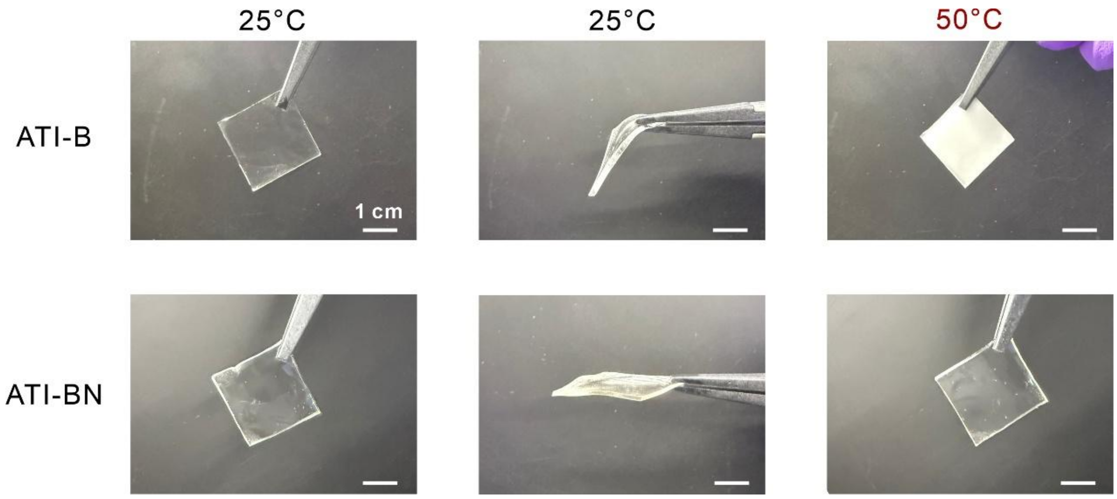

Supplementary Figure 21. Comparison of optical performance and mechanical appearance between ATI-B and ATI-BN under switching between 25°C and 50°C. Scale bar: 1 cm.

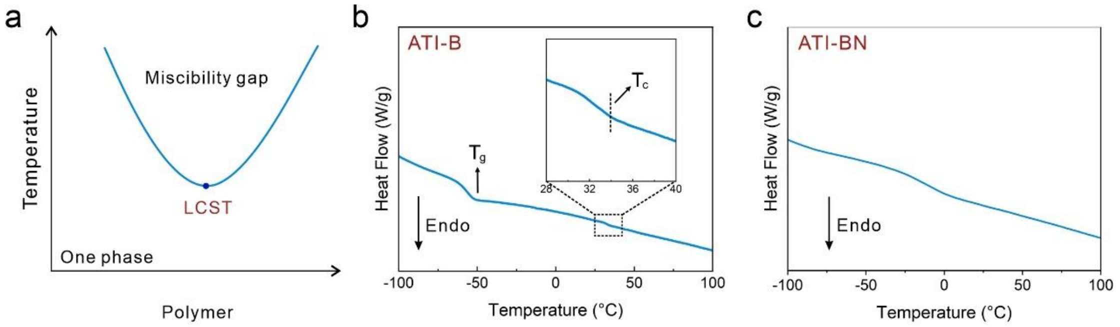

Supplementary Figure 22. LCST behavior in the temperature range of -100 to 100°C. a) LCST behavior of ATI-B. b) DSC curve of ATI-B and c) ATI-BN.

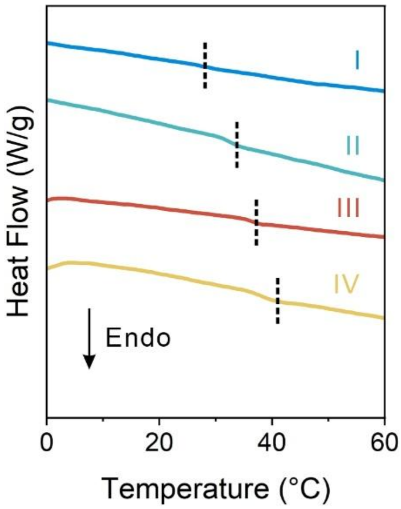

Supplementary Figure 23. DSC curves of ATI-B in different polymer/IL ratios.

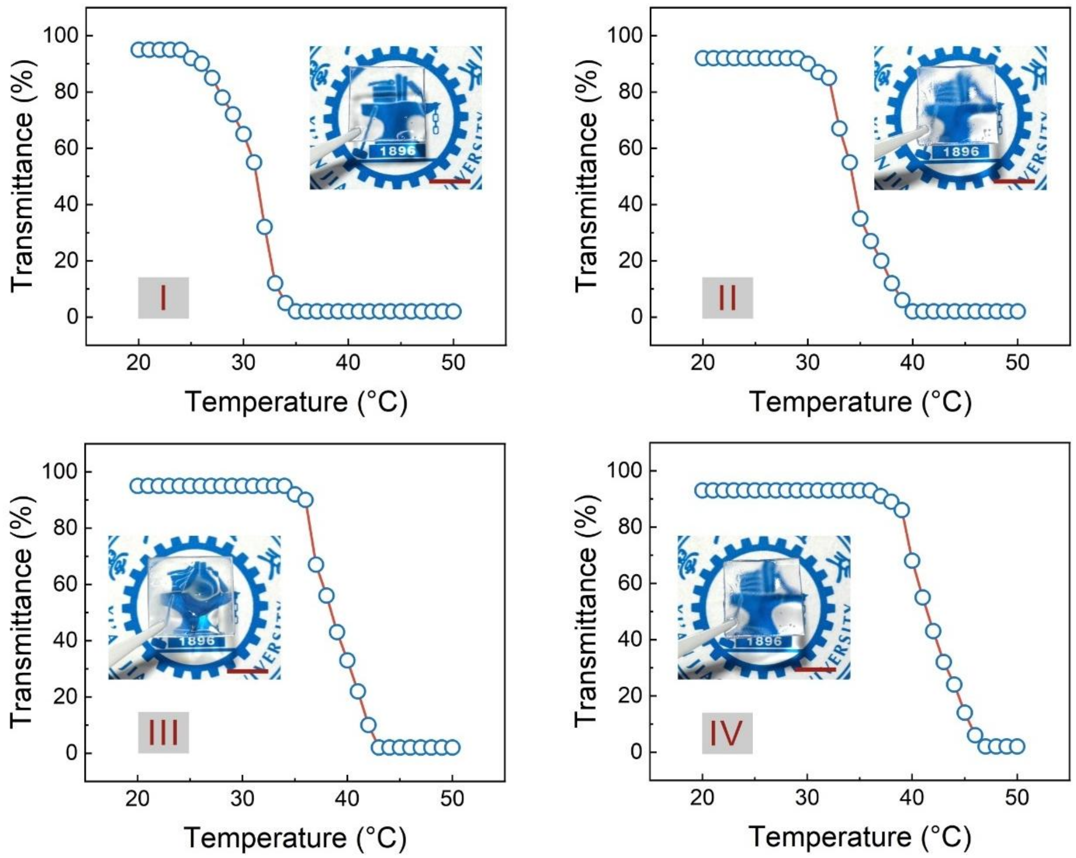

Supplementary Figure 24. Temperature dependence of transmittance at 550 nm for ATIs with different ATI-B formulations (see Supplementary Table 2 for compositions I-IV), insets showing transparency of ATIs at -70°C. Scale bar: 1 cm.

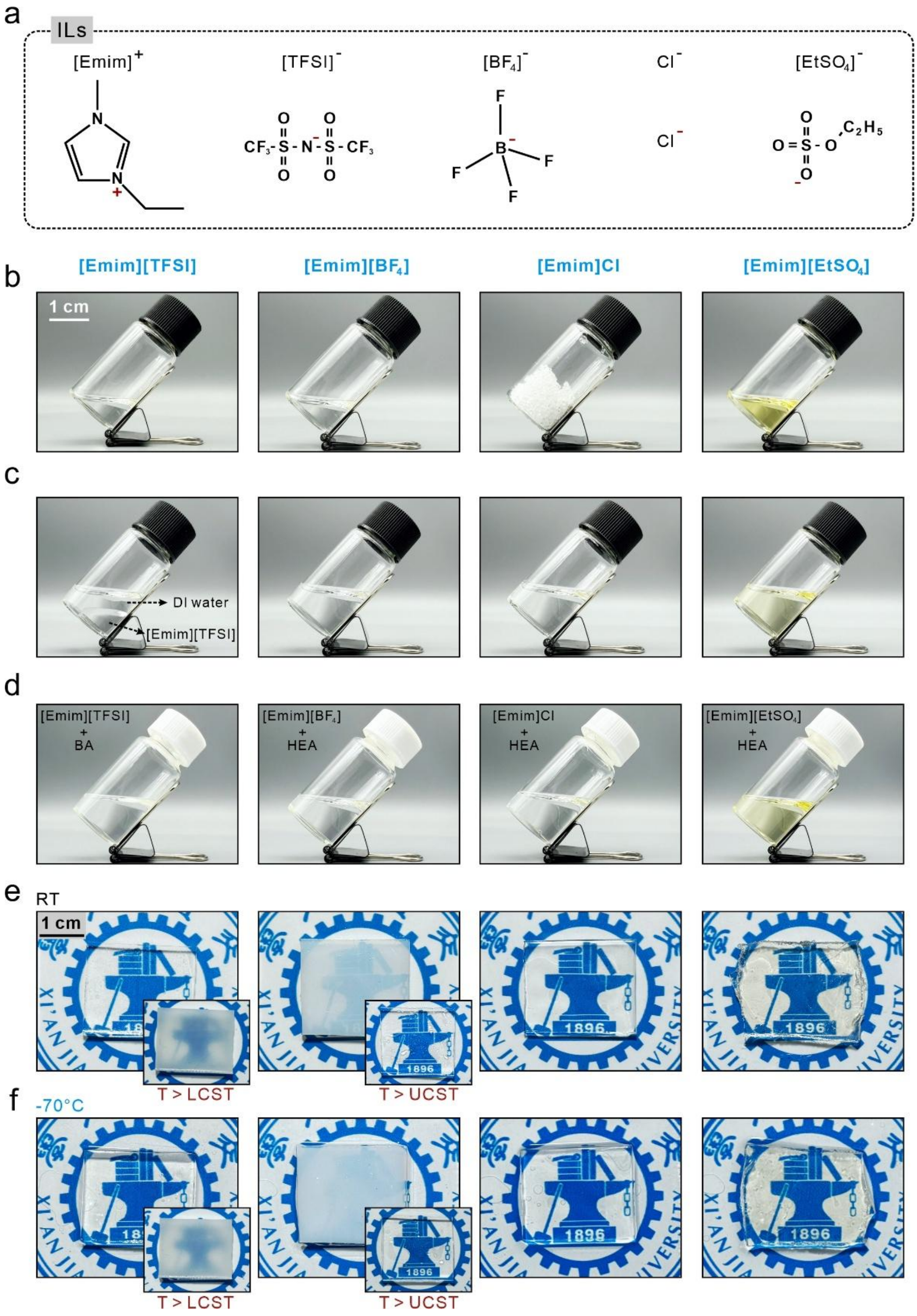

Supplementary Figure 25. Comparison of hydrophobicity and low-temperature tolerance among four commonly used ILs. a) The structures of the anions and cations of four ILs, where the cations are identical. b) Optical photographs of ILs. c) Demonstration of miscibility with water; IL:water = 1:1 wt/wt. d) Demonstration of precursor uniformity; IL:monomer = 1:1 wt/wt. e) Optical demonstration of ionogels. f) Optical demonstration of ionogels after cryogenic freezing at -70°C (achieved using a dry ice-ethanol bath).

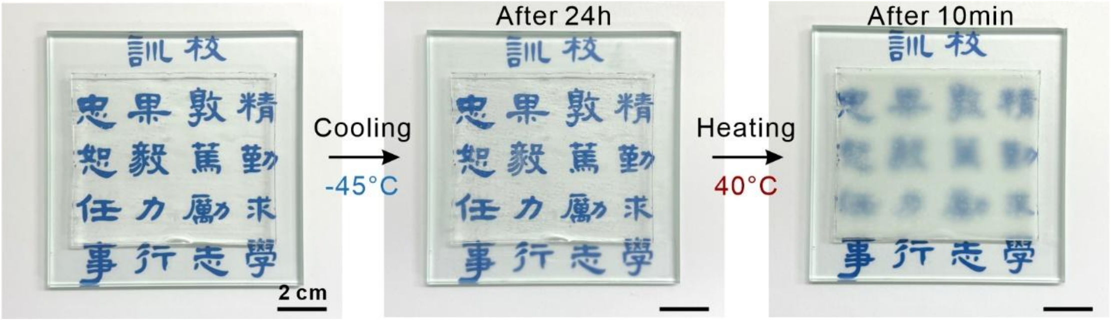

Supplementary Figure 26. Transparency changes of ATI-BN during freezing-heating process.

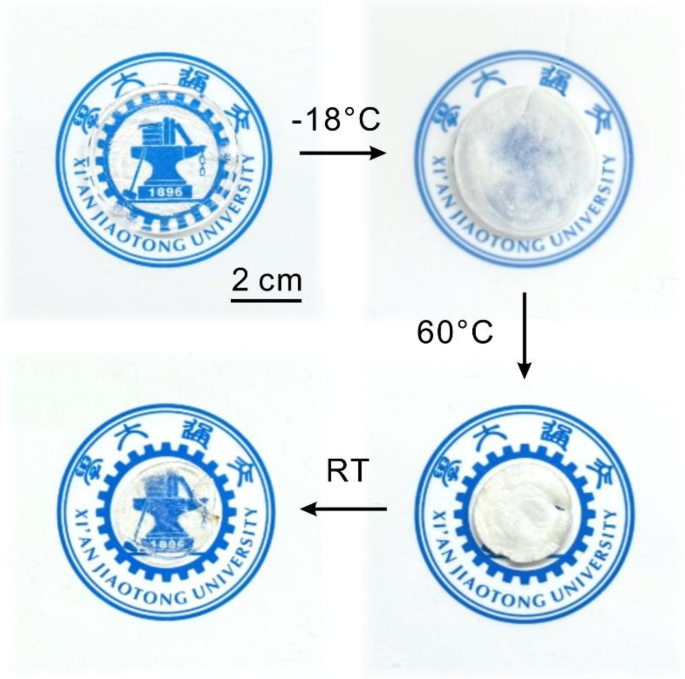

Supplementary Figure 27. Morphological changes in PNIPAM hydrogels during freezing-heating process.

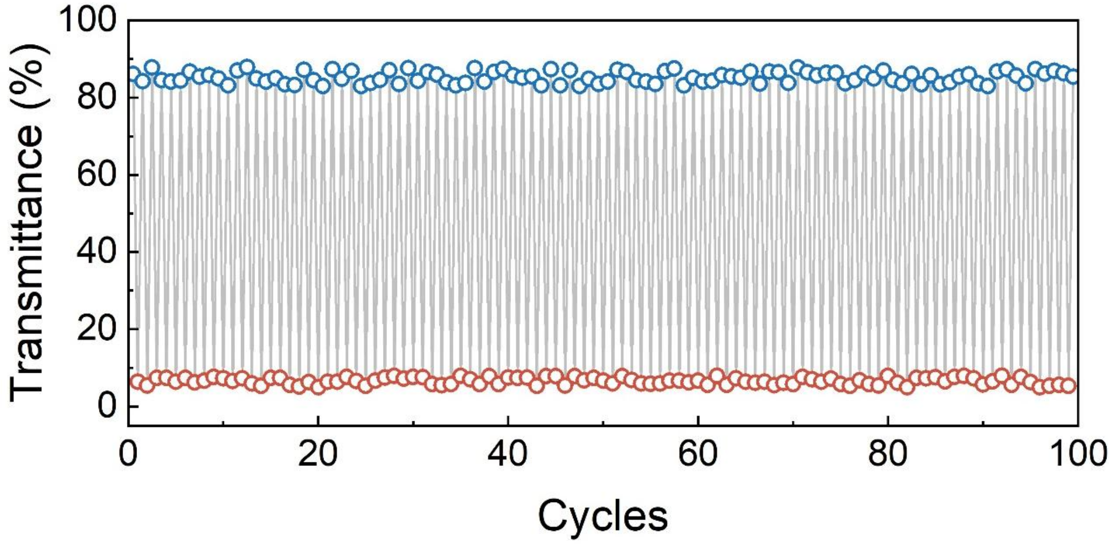

Supplementary Figure 28. Changes in transmittance (at 550 nm wavelength) of ATI over 100 cycles of freezing and heating from −45°C to 40°C.

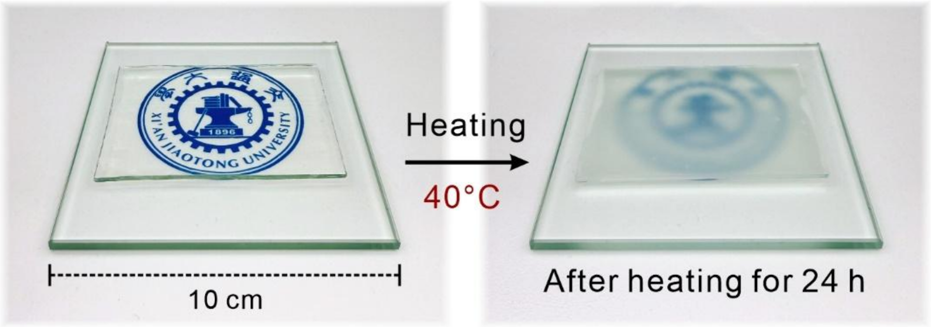

Supplementary Figure 29. Optical photographs of ATI before and after 24h continuous heating.

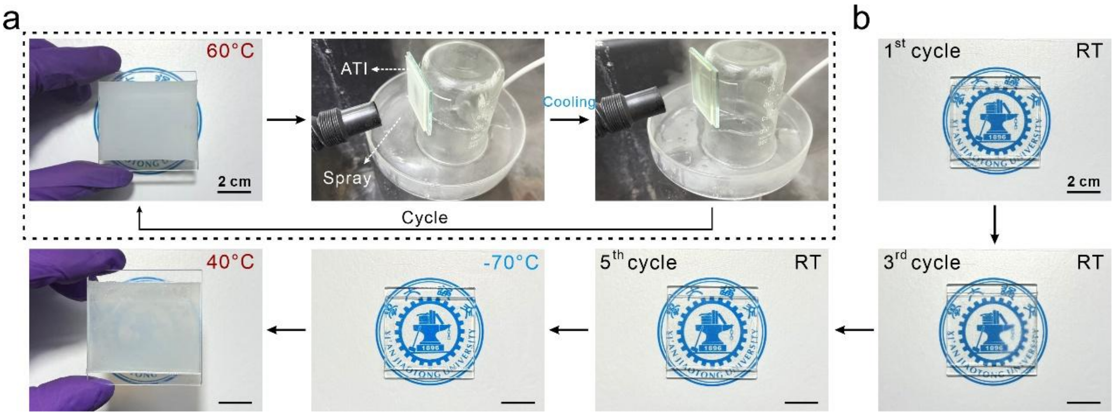

Supplementary Figure 30. High temperature and high humidity cyclic testing for ATI. a) High temperature and high humidity cyclic testing process. b) ATI optical photographs after each cycle, totaling 5 cycles.

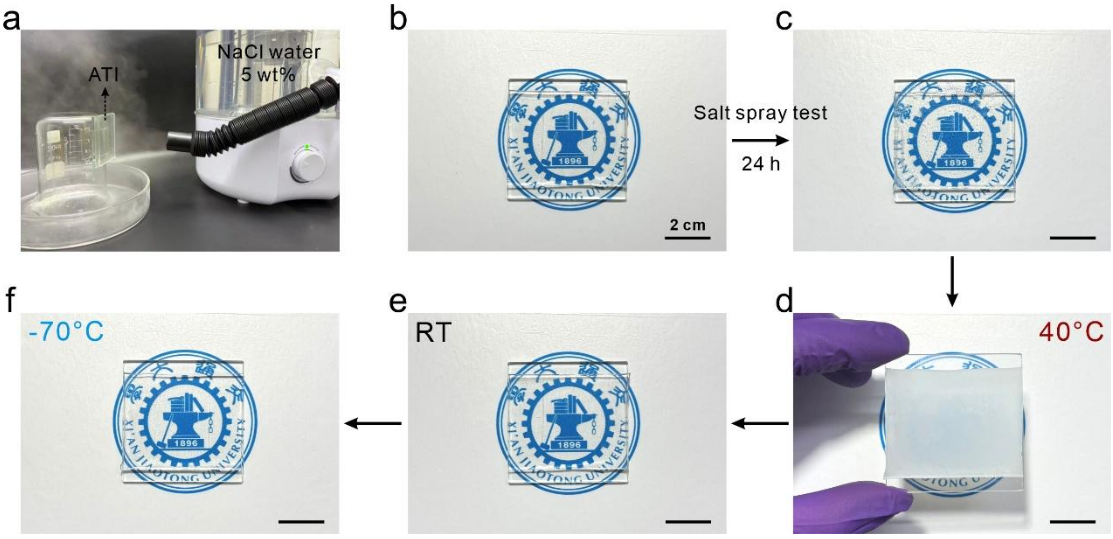

Supplementary Figure 31. Salt spray testing for the ATI. a) On-site photo of salt spray testing. b-f) Thermochromic performance of ATI after 24 h salt spray testing.

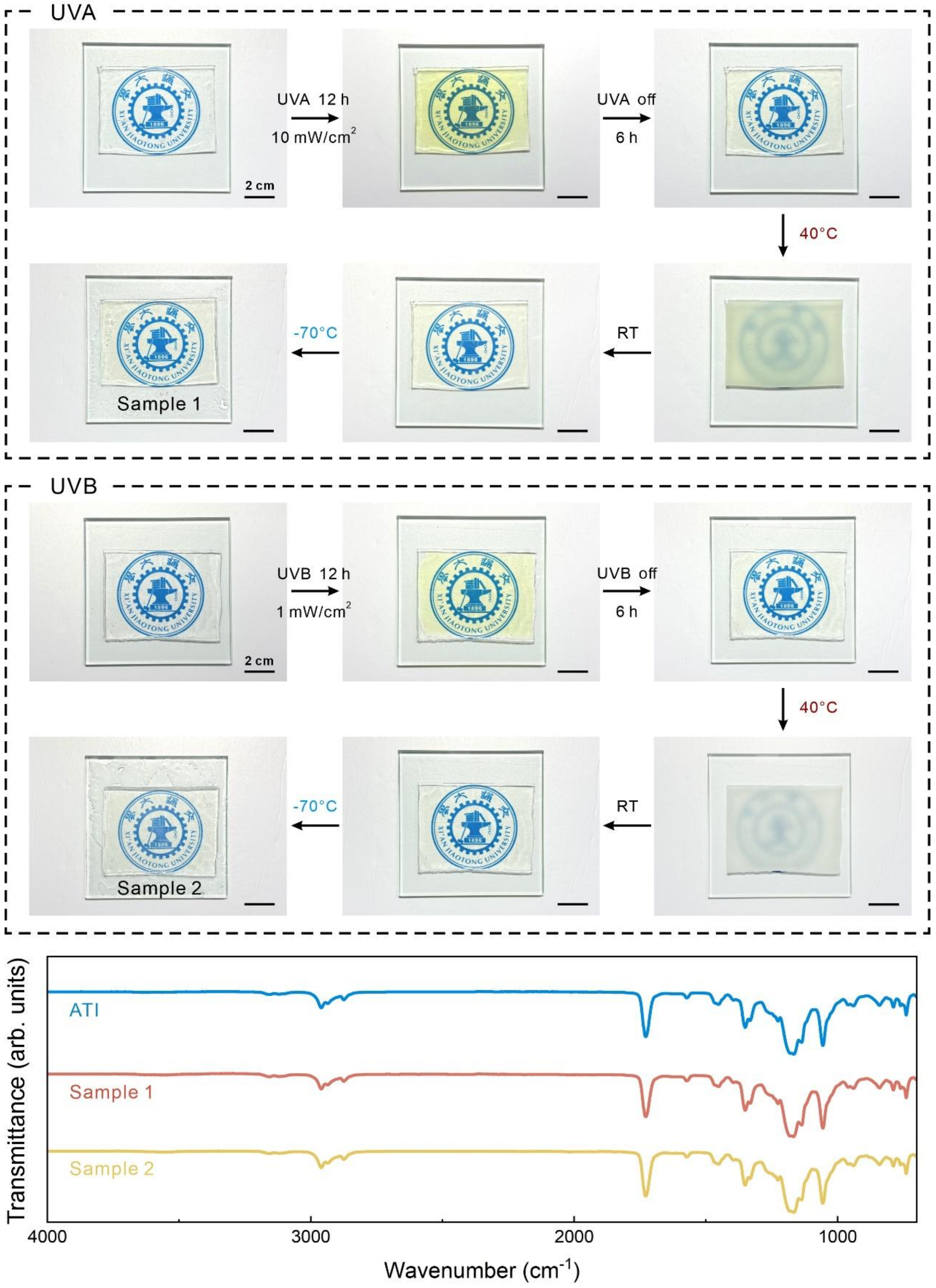

Supplementary Figure 32. Long-term stability of ATI under UVA and UVB irradiation, and FTIR spectra before and after irradiation.

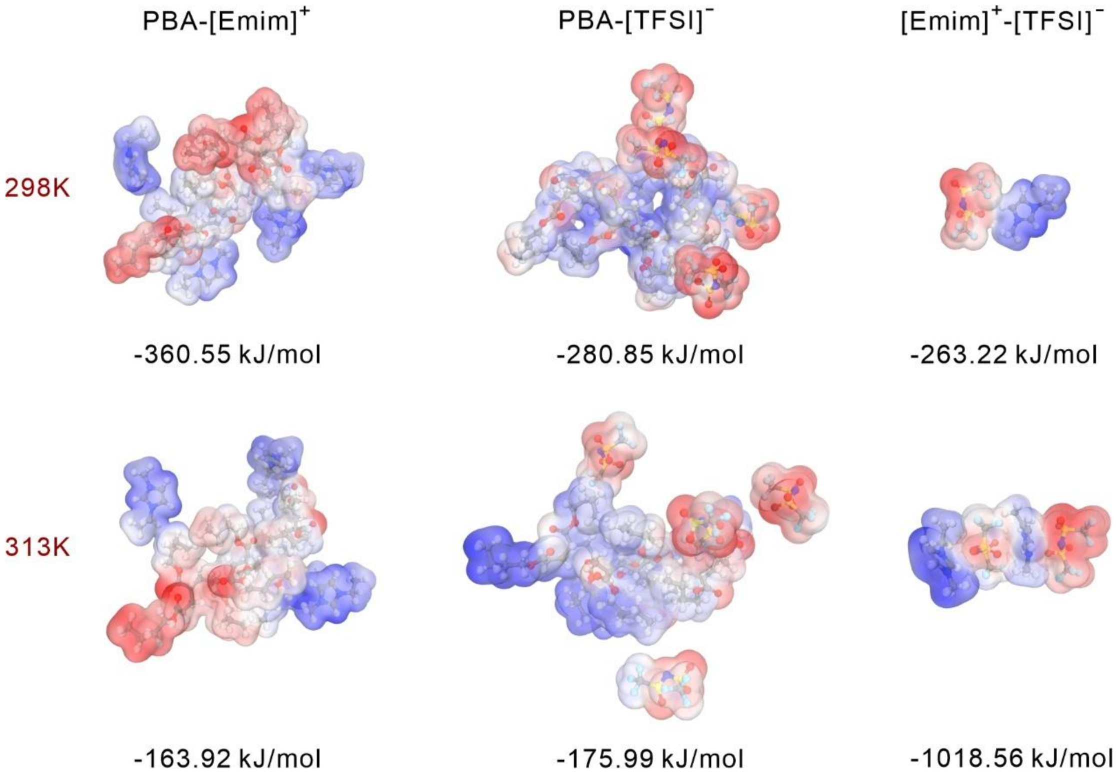

Supplementary Figure 33. Optimized molecular configurations and ESP simulations of the three main interactions at different temperatures in MD simulations.

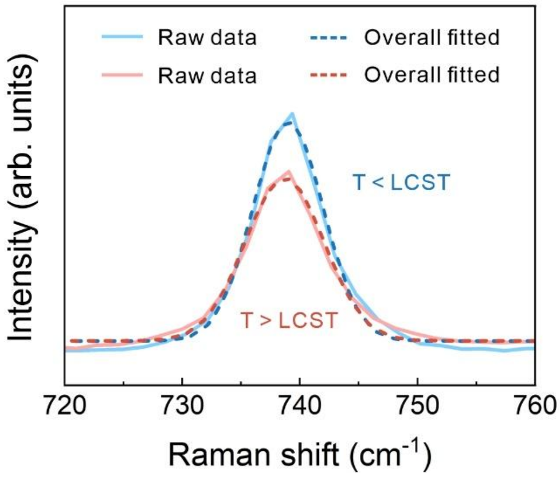

Supplementary Figure 34. Raman spectra at temperatures above and below the LCST, including fitted and raw data.

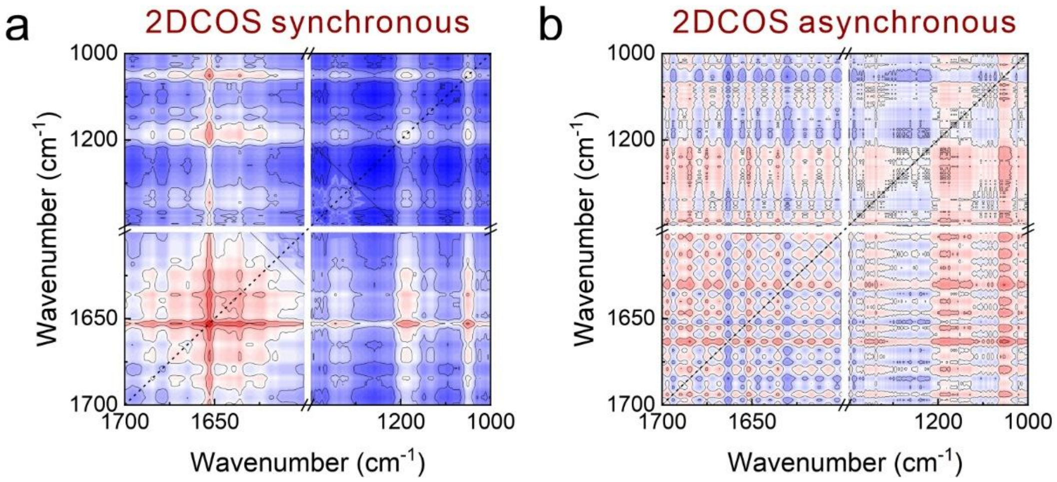

Supplementary Figure 35. 2D correlation spectra of temperature-dependent FTIR of ATI. a) 2DCOS synchronous spectra and b) 2DCOS asynchronous spectra. In 2DCOS spectra, the warm colors (red) represent positive intensities, while cold colors (blue) represent negative ones.

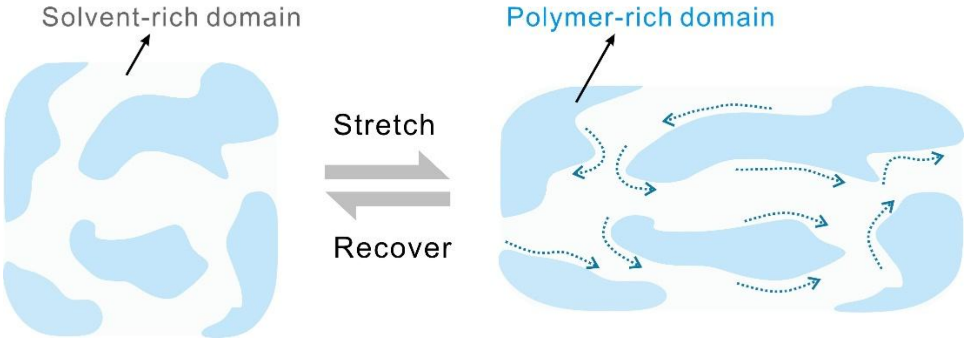

Supplementary Figure 36. Schematic of interphase load-transfer and energy-dissipation pathway in a phase-separated structure.

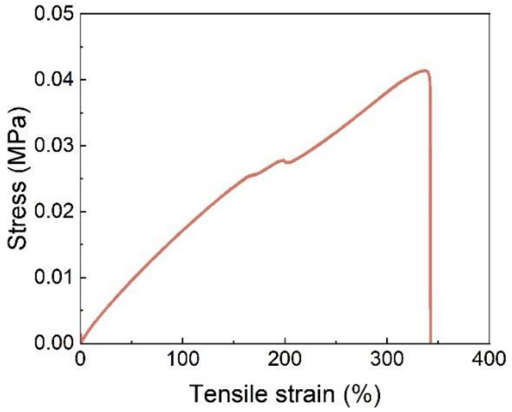

Supplementary Figure 37. Stress-strain curves of pure PBA ionogel.

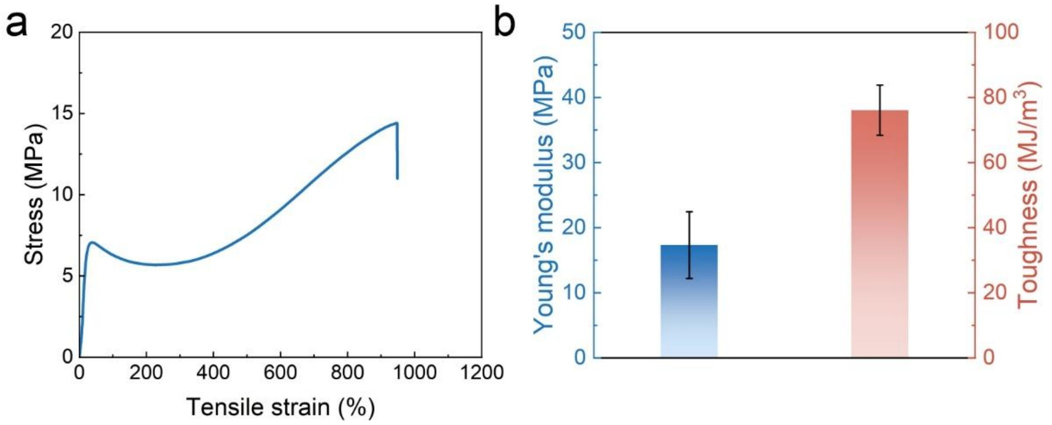

Supplementary Figure 38. Mechanical properties of pure P(BA-co-NIPAM) ionogel. a) Stress-strain curve. b) Young's modulus and toughness. Error bars represent standard deviation (n=3).

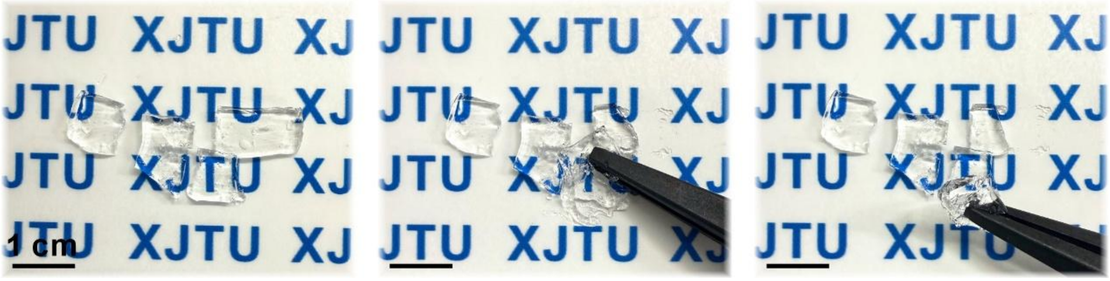

Supplementary Figure 39. Demonstration of weak mechanical properties of PNIPAM hydrogels.

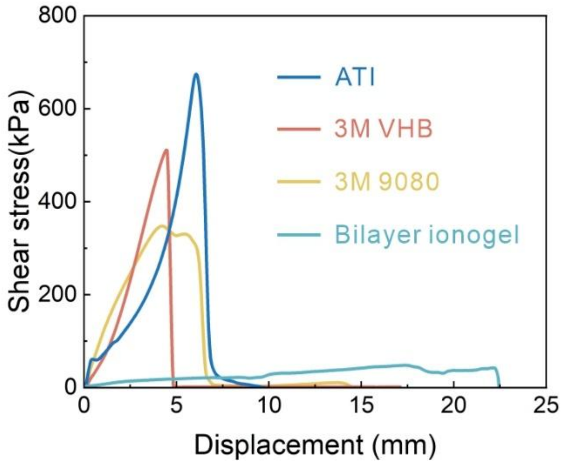

Supplementary Figure 40. Shear stress-displacement curve, including ATI, 3M VHB, 3M 9080, and bilayer ionogel.

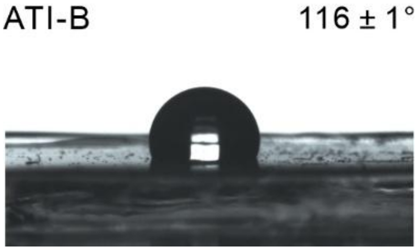

Supplementary Figure 41. Water contact angle image of ATI-B.

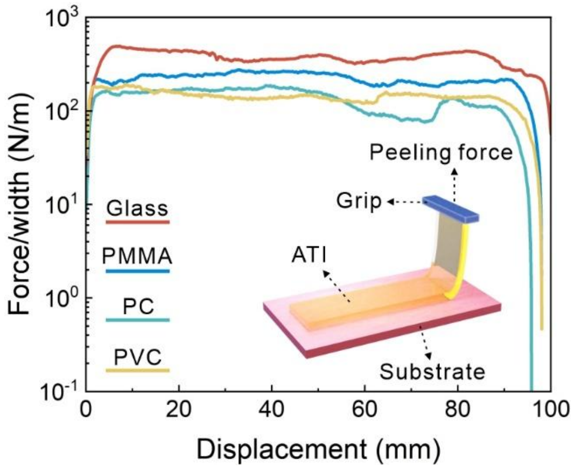

Supplementary Figure 42. The 90° peel strength of ATI on different substrates, with the insets showing the schematic of the testing method.

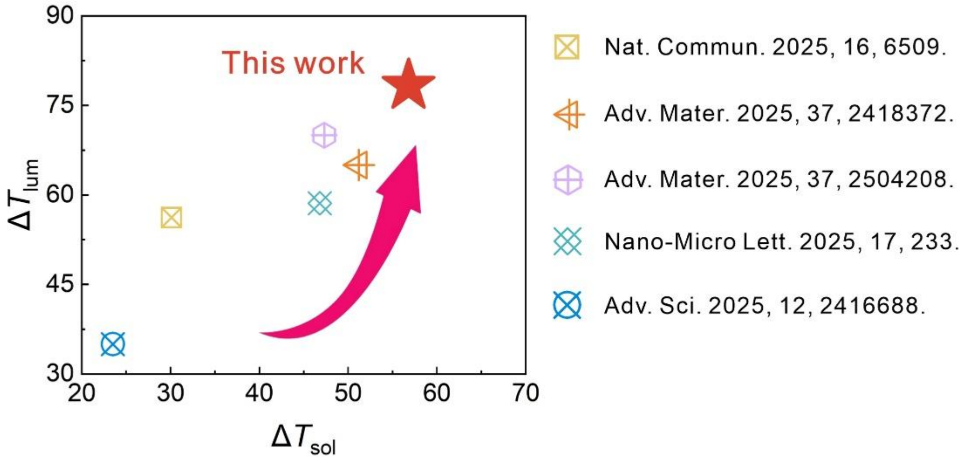

Supplementary Figure 43. Comparison of ATI's optical modulation capabilities (including ΔT_{lum} and ΔT_{sol}) with previously reported materials[6, 7, 8, 9, 10].

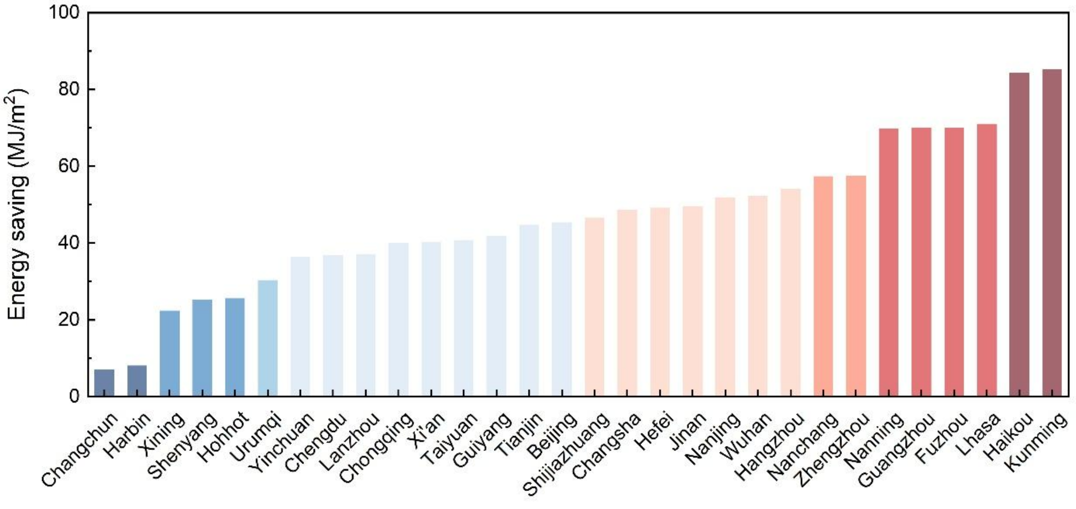

Supplementary Figure 44. Annual energy savings achieved by the ATI smart window model house in provincial capital cities across China through Energy Plus simulation.

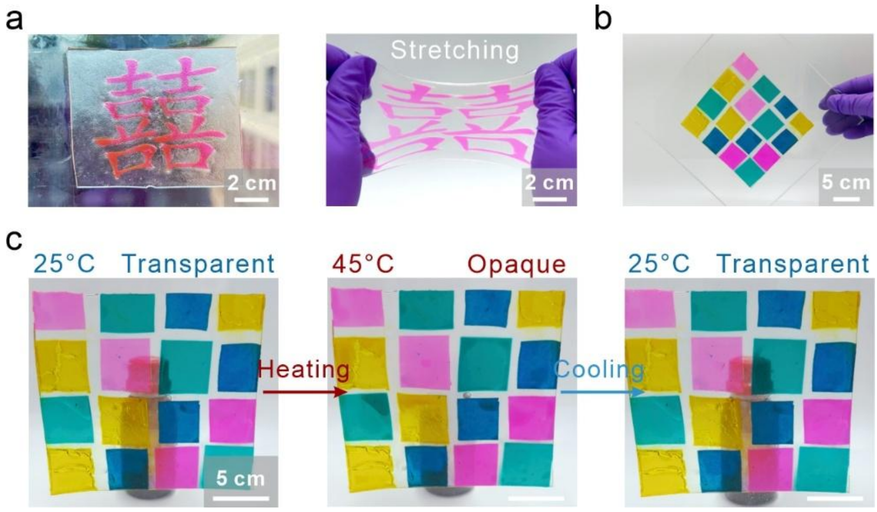

Supplementary Figure 45. Aesthetic customization by ATI. a) Custom display of the Chinese-style "Double Happiness" pattern on ATI, dyed with Rhodamine B. b) Modular coloring of ATI was performed using various dyes, including Rhodamine B, Soap Yellow, Toluidine Blue, and Brilliant Green. c) Demonstration of the thermochromic properties of dyed ATI.

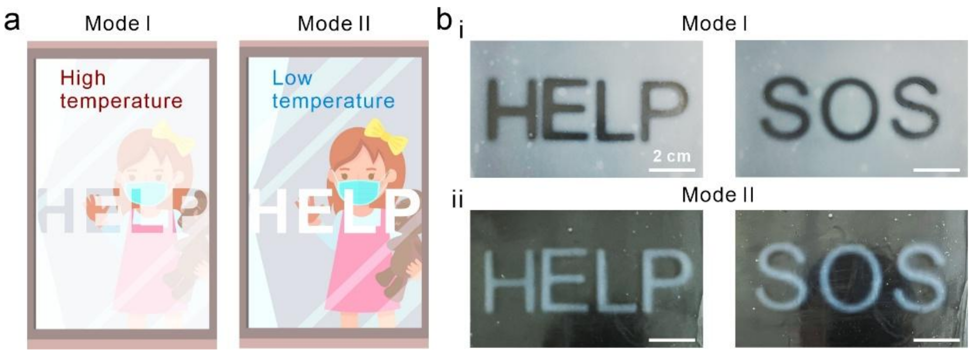

Supplementary Figure 46. Schematic diagram of information display using ATI-based smart display windows. a) Schematic for application in high and low temperature environments. b) Demonstration of application effects, including i) in high temperature environments (using cold objects as stamps) and ii) in low temperature environments (using hot objects as stamps).

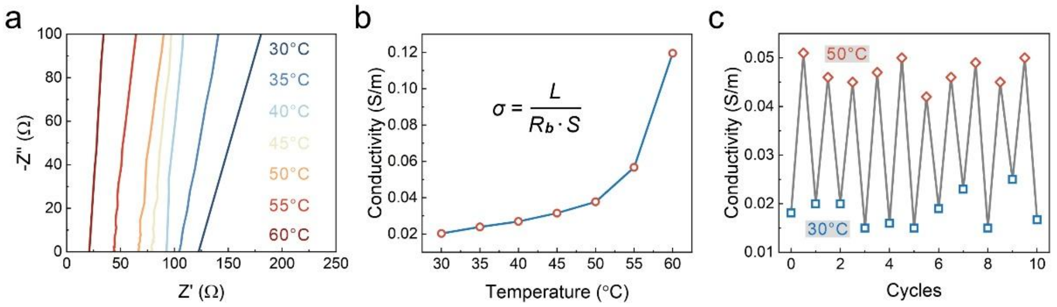

Supplementary Figure 47. Electrical conductivity test results. a) EIS curve. b) Curve of electrical conductivity versus temperature. c) Stability of electrical conductivity during heating-cooling cycles.

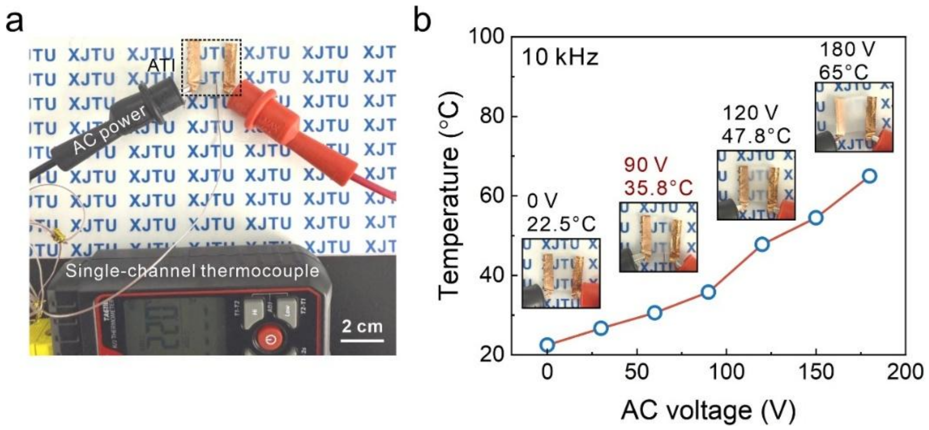

Supplementary Figure 48. The correlation between the surface temperature of ATI and the applied AC voltage. a) A photo from the test site shows the monitoring of ATI surface temperature using a single-channel T-type thermocouple. b) Relationship between ATI surface temperature and applied AC voltage; insets show optical photographs of ATIs at different voltages.

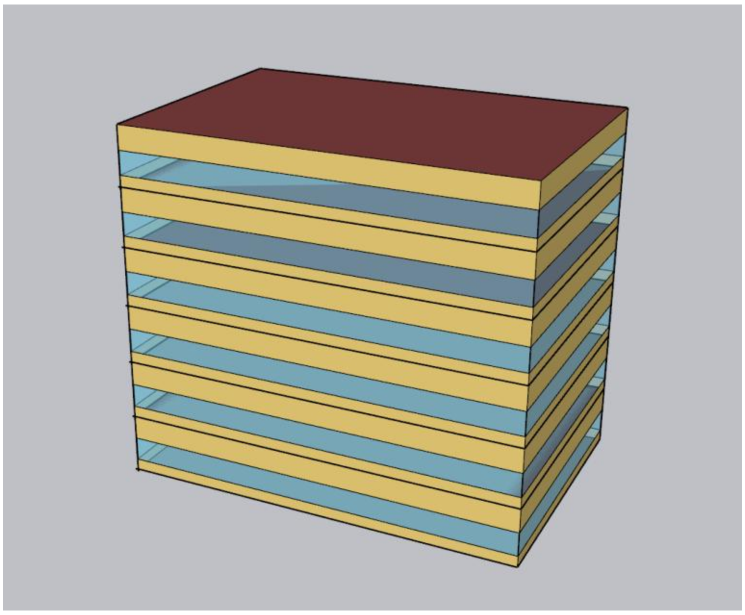

Supplementary Figure 49. Schematic of 27 m × 18 m × 4 m (6 stories) simplified houses for EnergyPlus simulation.

Supplementary Table 1. ***d*****-value in the variable-temperature WAXS of ATI-B.**

	20℃	30℃	40℃
d-1	7.7 Å	7.6 Å	7.2 Å
d-2	4.5 Å	4.5 Å	4.6 Å

Supplementary Table 2. Formulations of ATI-B with different LCSTs.

Group	Additions (Monomers in mmol)				LCST (℃)
	BA	[Emim][TFSI]	1173	EGDMA	
I	19.5	6.4	0.05 mol%	0.05 mol%	28
II	23.4	5.1			34
III	27.3	3.8			38
IV	31.2	2.5			41

Supplementary Table 3. Mechanical properties of ATI under different ATI-B formulations (see Supplementary Table 2), including tensile strength, toughness, and Young's modulus.

Group	I	II	III	IV
Tensile strength (MPa)	5.21	5.46	6.18	5.35
Toughness (MJ/m^3)	15.6	17.6	20.2	22.3
Young's modulus (MPa)	6.75	6.80	8.25	6.12

The comprehensive mechanical properties of ATI-BN significantly outperform those of ATI-B, making it the primary contributor to the mechanical performance of ATI (see Section 4). Consequently, fine-tuning the ATI-B formulation has not been observed to affect the mechanical behavior of ATI, including tensile strength, fracture toughness, and Young's modulus.

Supplementary Table 4. Multiplication results of the signs of each cross-peak in 2DCOS synchronous and asynchronous spectra (Supplementary Fig. 35) of the ATI-B.

1050	–	–	–	
1346	–	–		
1180	–			
1650				
	1650	1180	1346	1050

According to Noda's rule, the final specific order for ATI-B during heating is given as follows (→ means prior to or earlier than): v_{as}(-N-S) ([TFSI]$^-$, 1050 cm^{-1}) → v_{as}(-S=O) ([TFSI]$^-$, 1180 cm^{-1}) → v_s(-S=O) ([TFSI]$^-$, 1346 cm^{-1}) → v_{as}(-C=N) (imidazole ring of [Emim]$^+$, 1650 cm^{-1}).

Supplementary Table 5. Temperature-dependent T_{sol} and T_{lum} properties of ATI.

Temperature	T_{sol}	T_{lum}
20℃	75.7%	86.4%
45℃	18.9%	8.1%
Δ	56.8%	78.3%

Supplementary Note 1. FTIR analysis of ATIs.

With the introduction of PBA, the characteristic peaks in [Emim][TFSI] located at 1050 and 1347 cm^{-1} attributed to -N-S antisymmetric telescoping vibration and -S=O antisymmetric telescoping vibration, respectively, move to 1055 and 1351 cm^{-1}. Moreover, the characteristic peaks of -C-H symmetric stretching vibration and antisymmetric stretching vibration attributed to methyl group in PBA shift from 2873 and 2958 cm^{-1} to 2875 and 2960 cm^{-1}, respectively. All the blue shifts of the above characteristic peaks prove that strong ion-dipole interactions form between IL and the polymer (Fig. 2b)[11]. Similarly in ATI-BN, the formation of strong ion-dipole phase interactions is evidenced by the blue shifts of the characteristic peaks in the ionogel at 1058 and 1350 cm^{-1} attributed to the -N-S antisymmetric telescoping vibration and the -S=O antisymmetric telescoping vibration, as compared to the solvent-free P(BA-co-NIPAM). It is noteworthy that the occurrence of the red shift of the -C=O characteristic peak at 1175 cm^{-1} attributed to the ester group in the BA chain segment could be attributed to the increase in the bond length due to the steric effect in the phase-separated structure (Supplementary Fig. 10)[12].

Supplementary Note 2. LCST behavior of ATI-B.

The ATI proposed in this paper, particularly ATI-B, is a polymeric material with smart properties. The defining characteristic of thermoresponsive polymers lies in their response to temperature changes. Crucially, these polymers exhibit significant changes in solubility at their phase transition temperatures, primarily due to the precise compatibility window within their binary polymer/solvent phase diagrams. Based on their thermoresponsive behavior, these polymers are primarily categorized into two types: low critical solution temperature (LCST) behavior and high critical solution temperature (UCST) behavior. LCST behavior implies that the polymer remains soluble in the solvent before reaching the phase transition temperature. Beyond this temperature, two distinct phases emerge—a diluted polymer phase and a concentrated polymer phase. Essentially, LCST characterizes the lowest point of phase diagram for a miscible system, beyond which only a single phase can exist, irrespective of polymer concentration (Supplementary Fig. 22a)[13]. In ionogel systems, the LCST phenomenon arises from the reversible interactions between the polymer and the solvent, governing the solvation state of the polymer. Differential scanning calorimetry (DSC) is a commonly used method for determining the LCST of thermosensitive polymers, as their phase transitions are often accompanied by endothermic events[14]. DSC measurements were conducted on ATI-B and ATI-BN. The results indicated that, besides a step-like shift in the baseline, ATI-B displayed an additional endothermic peak near 34 °C, which was absent in ATI-BN. The step-like shift corresponds to the glass transition temperature (T_g) of the polymer. Within this temperature region, the specific heat capacity of the polymer increases, leading to greater heat absorption and a shift of the baseline toward the endothermic direction. The weak endothermic peak is ascribed to the LCST behavior of the polymer (Supplementary Fig. 22b and 22c). At this temperature, the interactions between the polymer and the IL solvent are disrupted, leading to precipitation of the polymer from the solvent. This process is endothermic, producing a small enthalpy change. This is because in ATI-B, the dynamic variation in ion-dipole interactions between the polymer and ionic liquid is insufficiently large to be classified

as a large-scale phase separation phenomenon accompanied by significant solvent expulsion (with large enthalpy change). Consequently, the endothermic peak on the DSC curve is not prominent, which is consistent with previous reports[15]. Moreover, a key characteristic of LCST behavior is a significant alteration in the material's light-scattering capability, aligning with our observations in visible-wavelength transmittance tests (Supplementary Fig. 18).

Supplementary Note 3. Hydrophobicity and low-temperature resistance of common imidazolium ILs.

To investigate the correlation between hydrophobicity, volatility, melting point, and low-temperature tolerance of imidazolium ILs, four ILs sharing the common 1-ethyl-3-methylimidazolium ($[Emim]^+$) cation but differing anions were selected: [Emim][TFSI], $[Emim][BF_4]$, [Emim]Cl, and $[Emim][EtSO_4]$ (Supplementary Fig. 25a). The anion variation imparts distinct hydrophobicity, hygroscopicity, and thermodynamic properties. Their melting points are approximately -18 °C, 15 °C, 84 °C, and -37 °C, respectively. All exhibit negligible vapor pressure at elevated temperatures. At room temperature, [Emim]Cl is solid and highly hygroscopic due to its chloride anion (Supplementary Fig. 25b). Hydrophilicity follows the order: [Emim][TFSI] < $[Emim][BF_4]$ < [Emim]Cl < $[Emim][EtSO_4]$, while hydrophobicity shows the opposite trend. Hydrophobicity was confirmed by water miscibility: [Emim][TFSI] is completely immiscible (most hydrophobic), whereas the other three are miscible (Supplementary Fig. 25c).

For monomer selection, the hydrophobic monomer butyl acrylate (BA) is compatible with hydrophobic [Emim][TFSI], while the more hydrophilic ionic liquids ($[Emim][BF_4]$, [Emim]Cl, $[Emim][EtSO_4]$) show better compatibility with the hydrophilic monomer 2-hydroxyethyl acrylate (HEA) (Supplementary Fig. 25d). The crosslinker and photoinitiator were consistent with those used in this work. After the same low-temperature UV curing process, four distinct ionogels were obtained (Supplementary Fig. 25e). Similar to the high-performing [Emim][TFSI]/BA system, the $[Emim][BF_4]$/HEA combination exhibited reversible upper critical solution temperature (UCST) behavior, turning transparent at high temperatures and opaque at low temperatures. In contrast, ionogels based on [Emim]Cl and $[Emim][EtSO_4]$ showed no observable thermosensitive phase transition. Remarkably, after identical low-temperature treatment, all four ionogels demonstrated excellent low-temperature tolerance while fully retaining their respective phase transition characteristics (Supplementary Fig. 25f). Even at the experimental lower limit of -70 °C, all ionogels

maintained excellent performance.

This low-temperature tolerance is primarily attributed to the inherent properties of imidazolium ILs. Their structural asymmetry, arising from bulky, asymmetric ions, inhibits regular crystalline packing upon cooling, suppressing crystallization and leading to low melting points and a propensity for supercooling. Furthermore, physical interactions (e.g., ion-dipole) between the ILs and polymer chains are believed to further inhibit IL crystallization, thereby enhancing the low-temperature tolerance of the ionogels[16].

Supplementary Note 4. Two-dimensional correlation spectroscopy (2DCOS).

Two-dimensional correlation spectroscopy (2DCOS) is an analytical technique originally proposed by Noda[17, 18]. It facilitates the interpretation of spectroscopic intensity variations induced by various external perturbations, including time, temperature, concentration, pressure, and electric field strength[19, 20]. By spreading spectral peaks over two dimensions, 2Dcos resolves spectral overlap and enhances spectral resolution.

A 2D correlation map is characterized by two independent wavenumber axes (ν_1, ν_2) and a correlation intensity axis. Analyses typically yield two types of spectra: synchronous and asynchronous. The correlation intensities in these maps reflect whether spectral changes occur in-phase (synchronous) or out-of-phase (asynchronous) relative to each other.

Synchronous Spectrum: This spectrum represents simultaneous or coincidental intensity changes at wavenumbers ν_1 and ν_2 in response to the external perturbation. The synchronous spectrum is symmetric across its diagonal. Peaks appearing on the diagonal are termed autopeaks. Their intensity is always positive, representing the autocorrelation magnitude of perturbation-induced molecular vibrations at each specific wavenumber. Significant autopeaks indicate regions where spectral intensity changes prominently under perturbation. Peaks appearing off the diagonal are termed cross-peaks. They can be either positive or negative. A positive cross-peak indicates that the intensity changes at ν_1 and ν_2 occur in the same direction (both increase or both decrease) during the perturbation. Conversely, a negative cross-peak signifies that the intensity changes at ν_1 and ν_2 occur in opposite directions (one increases while the other decreases).

Asynchronous Spectrum: This spectrum depicts sequential or temporally unsynchronized intensity changes at ν_1 and ν_2. Unlike the synchronous spectrum, the asynchronous spectrum is asymmetric with respect to its diagonal and contains only off-diagonal cross-peaks, which can be positive or negative. Asynchronous cross-peaks develop exclusively when the dynamic intensity variations at ν_1 and ν_2 are out of phase

(e.g., one delayed or accelerated relative to the other). Crucially, these peaks appear only if ν_1 and ν_2 arise from different molecular sources or functional groups experiencing distinct effects due to the perturbation. Thus, asynchronous cross-peaks are particularly powerful for resolving overlapped bands originating from different chemical species, phases, or functional groups within dissimilar molecular environments.

Supplementary Note 5. Entropy-driven dynamic phase separation in ATI-B.

Numerous studies report that hydrogen bonding and cation-π interactions can induce ordered structures between ILs and polymers, thereby generating a negative mixing entropy (ΔS_{mix}). Similarly, we propose that oriented solvation driven by ion-dipole interactions in our system could serve as the driving force for the observed LCST behavior. At room temperature, the Gibbs free energy of mixing ($\Delta G_{mix} = \Delta H_{mix} - T\Delta S_{mix}$) is negative. However, heating endothermically disrupts these ion-dipole interactions, making ΔH_{mix} less negative. Concurrently, the system gains entropy, increasing the magnitude of the entropic contribution ($T|\Delta S_{mix}|$). When $T|\Delta S_{mix}|$ exceeds $|\Delta H_{mix}|$, ΔG_{mix} becomes positive. This results in entropy-driven phase separation, where the ILs segregate into discrete domains.

Supplementary Note 6. Calculation of transmittance and modulation capability.

The T_{lum} and T_{sol} are calculated by the following equation[21]:

$$T_{lum,sol} = \frac{\int \varphi_{lum,sol}(\lambda) T(\lambda) d(\lambda)}{\int \varphi_{lum,sol}(\lambda) d(\lambda)} \quad (4)$$

where, the φ_{lum} is the luminous vision spectral photo efficiency of the human eye (380-780 nm), φ_{sol} is AM 1.5 standard solar irradiation spectrum (300-2500 nm), $T(\lambda)$ is the spectral transmittance. Where φ_{lum} uses the CIE spectral luminous efficiency for photopic vision covering 380-780 nm and φ_{sol} uses the ASTM G173-03 standard spectrum covering 300-2500 nm.

The ΔT_{lum} and ΔT_{sol} are calculated by the following equation:

$$\Delta T_{lum,sol} = \Delta T_{lum,sol}(Transparent) - \Delta T_{lum,sol}(Opaque) \quad (5)$$

References

S1. Grimme S, Antony J, Ehrlich S, Krieg H. A consistent and accurate ab initio parametrization of density functional dispersion correction (DFT-D) for the 94 elements H-Pu. *The Journal of Chemical Physics* **132**, 154104 (2010).

S2. Perdew JP, Ernzerhof M, Burke K. Rationale for mixing exact exchange with density functional approximations. *The Journal of Chemical Physics* **105**, 9982-9985 (1996).

S3. Perdew JP, Burke K, Ernzerhof M. Generalized Gradient Approximation Made Simple. *Physical Review Letters* **77**, 3865-3868 (1996).

S4. Zhang P*, et al.* Biomimetic Superhydrophobic Triboelectric Surface Prepared by Interfacial Self-Assembly for Water Harvesting. *Advanced Functional Materials* **35**, 2413201 (2025).

S5. Dupré A, Dupré P. *Théorie mécanique de la chaleur*. Paris : Gauthier-Villars (1869).

S6. Wang H*, et al.* Hydrated ionic polymer for thermochromic smart windows in buildings. *Nature Communications* **16**, 6509 (2025).

S7. Wang K*, et al.* Hofmeister Effect-Enhanced, Nanoparticle-Shielded, Thermally Stable Hydrogels for Anti-UV, Fast-Response, and All-Day-Modulated Smart Windows. *Advanced Materials* **37**, 2418372 (2025).

S8. Mo S, Gao L. All-Season Thermochromic Window With Customizable Optical Switching Temperature and Directional Thermal Radiation. *Advanced Materials* **37**, 2504208 (2025).

S9. Zhang Z*, et al.* A Janus Smart Window for Temperature-Adaptive Radiative Cooling and Adjustable Solar Transmittance. *Nano-Micro Letters* **17**, 233 (2025).

S10. Liu M*, et al.* Three-State Thermochromic Smart Window for Building Energy-Saving. *Advanced Science* **12**, 2416688 (2025).

S11. Huang H, Sun L, Zhang Y, Zhang R, Chen C, Sun W. Ionogels Reinforced by Ionophobic Coordination. *Advanced Materials* **37**, 2506563 (2025).

S12. Yin J*, et al.* Theoretical insights into CO_2/N_2 selectivity of the porous ionic liquids

constructed by ion-dipole interactions. *Journal of Molecular Liquids* **344**, 117676 (2021).

S13. Zhou Q, Men Y. Thermoresponsive ionogels. *Polymer Chemistry* **15**, 2719-2739 (2024).

S14. Aravopoulou D, Kyriakos K, Miasnikova A, Laschewsky A, Papadakis CM, Kyritsis A. Comparative Investigation of the Thermoresponsive Behavior of Two Diblock Copolymers Comprising PNIPAM and PMDEGA Blocks. *The Journal of Physical Chemistry B* **122**, 2655-2668 (2018).

S15. Liu Y*, et al.* A Stable and Self-Healing Thermochromic Polymer Coating for All Weather Thermal Regulation. *Advanced Functional Materials* **33**, 2307240 (2023).

S16. Sun L*, et al.* Ionogel-based, highly stretchable, transparent, durable triboelectric nanogenerators for energy harvesting and motion sensing over a wide temperature range. *Nano Energy* **63**, 103847 (2019).

S17. Noda I. Two-dimensional infrared spectroscopy. *Journal of the American Chemical Society* **111**, 8116-8118 (1989).

S18. Noda I. Generalized Two-Dimensional Correlation Method Applicable to Infrared, Raman, and Other Types of Spectroscopy. *Appl Spectrosc* **47**, 1329-1336 (1993).

S19. Sun S-t, Wu P-y. Spectral insights into microdynamics of thermoresponsive polymers from the perspective of two-dimensional correlation spectroscopy. *Chinese Journal of Polymer Science* **35**, 700-712 (2017).

S20. Noda I. Recent advancement in the field of two-dimensional correlation spectroscopy. *Journal of Molecular Structure* **883-884**, 2-26 (2008).

S21. Li B, Xu F, Guan T, Li Y, Sun J. Self-Adhesive Self-Healing Thermochromic Ionogels for Smart Windows with Excellent Environmental and Mechanical Stability, Solar Modulation, and Antifogging Capabilities. *Advanced Materials* **35**, 2211456 (2023).
