# Supplementary material for: Tough asymmetric thermochromic ionogels via dynamic in situ phase separation for dual-modal smart optical switching
Source: Nat Commun. 2026 Mar 18;17:4124. doi: 10.1038/s41467-026-70830-4 (PMC13150026; doi:10.1038/s41467-026-70830-4)
Supplement: Supplementary file 2 — Description of Additional Supplementary Files [file 41467_2026_70830_MOESM2_ESM.pdf]

Supplementary Movie 1: Thermochromic performance of ATIs

Supplementary Movie 2: Demonstration of the transparency of ATls at -196°C

Supplementary Movie 3: The thermosensitive dynamic phase separation process in ATI-B

Supplementary Movie 4: Demonstration of the transparency of ATls at -70°C

Supplementary Movie 5: Demonstration of tough mechanical performance of ATls

Supplementary Movie 6: Surface hydrophobicity and antifouling property of ATls

Supplementary Movie 7: Thermochromism mediated by in situ Joule heating of ATls

Supplementary Movie 8: Demonstration of ATl-based localized optical switch projection
